# Hot Vibrational States in a High-Performance Multiple Resonance Emitter and the Effect of Excimer Quenching on Organic Light-Emitting Diodes

**DOI:** 10.1021/acsami.0c20619

**Published:** 2021-02-08

**Authors:** Kleitos Stavrou, Andrew Danos, Toshiki Hama, Takuji Hatakeyama, Andrew Monkman

**Affiliations:** † Department of Chemistry, School of Science and Technology, Kwansei Gakuin University, 2-1 Gakuen, Sanda, Hyogo 669-1337, Japan; ‡ Department of Physics, Durham University, South Road, Durham, DH1 3LE, United Kingdom

**Keywords:** OLEDs, multiple resonance emitter, photophysics, excimer, hyperfluorescence, FRET

## Abstract

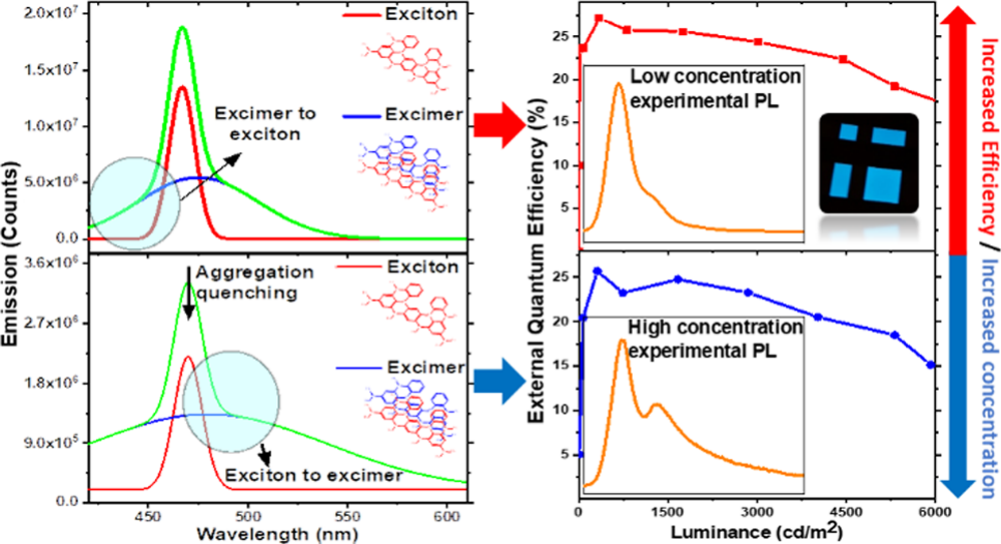

The photophysics of multiple resonance thermally activated delayed fluorescence molecule ν-DABNA is described. We show coupling of a 285 cm^–1^ stretching/scissoring vibrational mode of peripheral phenyl rings to the S_1_ state, which dictates the ultimate emission full-width at half maximum. However, a separate high amplitude mode, 945 cm^–1^ of the *N*-biphenyl units, mediates the reverse intersystem crossing (rISC) mechanism. Concentration-dependent studies in solution and solid state reveal a second emission band that increases nonlinearly with concentration, independent of the environment assigned to excimer emission. Even at concentrations well below those used in devices, the excimer contribution affects performance. Using different solvents and solid hosts, rISC rates between 3–6 × 10^5^ s^–1^ are calculated, which show negligible dependence on environmental polarity or host packing. At 20 K over the first 10 ns, we observe a broad Gaussian excimer emission band with energy on-set above the S_1_ exciton band. An optical singlet-triplet gap (ΔE_ST_) of 70 meV is measured, agreeing with previous thermal estimates; however, the triplet energy is also found to be temperature-dependent. A monotonic increase of the exciton emission band full-width at half maximum with temperature indicates the role of hot transitions in forming vibrational excited states at room temperature (RT), and combined with an observed temperature dependency of ΔE_ST_, we deduce that the rISC mechanism is that of thermally activated reverse internal conversion of T_1_ to T*_N_* (*n* ≥ 2) followed by rapid rISC of T*_N_* to S_1_. Organic light-emitting diodes with ν-DABNA as a hyperfluorescent emitter (0.5 wt % and 1 wt %) exhibit an increase of maximum external quantum efficiency, reaching 27.5% for the lower ν-DABNA concentration. On the contrary, a Förster radius analysis indicated that the energy transfer ratio is smaller because of higher donor–acceptor separation (>2.4 nm) with weak sensitizer emission observed in the electroluminescence. This indicates excimer quenching in 1 wt % devices.

## Introduction

Thermally activated delayed fluorescence (TADF) materials have attracted significant attention as rare earth metal free emitters, which can realize organic light-emitting diodes (OLEDs) with an internal quantum efficiency of 100%.^1,2^ Such systems require a small singlet-triplet energy gap (Δ*E*_ST_) to facilitate spin upconversion from the lowest triplet excited state (T_1_) to the lowest singlet excited state (S_1_). The common strategy to reduce Δ*E*_ST_ is to use electronically decoupled donor and acceptor groups that give rise to spatial separation of the highest occupied molecular orbital (HOMO) and the lowest occupied molecular orbital (LUMO).^3^ However, donor–acceptor (D–A) materials give rise to broadband charge transfer emission spectra with a full-width at half maximum (FWHM) of 70–100 nm because of structural relaxation in S_1_, which has hampered the practical application of TADF materials in OLED displays.

To overcome this drawback, we have reported an alternative strategy for HOMO–LUMO separation featuring multiple resonance (MR) effects of boron and nitrogen atoms.^4−7^ This effect localizes HOMOs and LUMOs on different atoms of a single aromatic system to realize not only small Δ*E*_ST_ (<200 meV) but also narrowband emission (FWHM of <30 nm) and high photoluminescence quantum yield (PLQY). These attractive characteristics have encouraged our group^8−11^ and others^12−17^ to develop a variety of MR-TADF materials. However, all these materials exhibit relatively small reverse intersystem crossing (rISC) rates (*k*_rISC_; ∼10^4^ s^–1^) and consequently suffer from efficiency roll-off at high luminance because of their slow spin upconversion mechanism.

**Figure 1:**
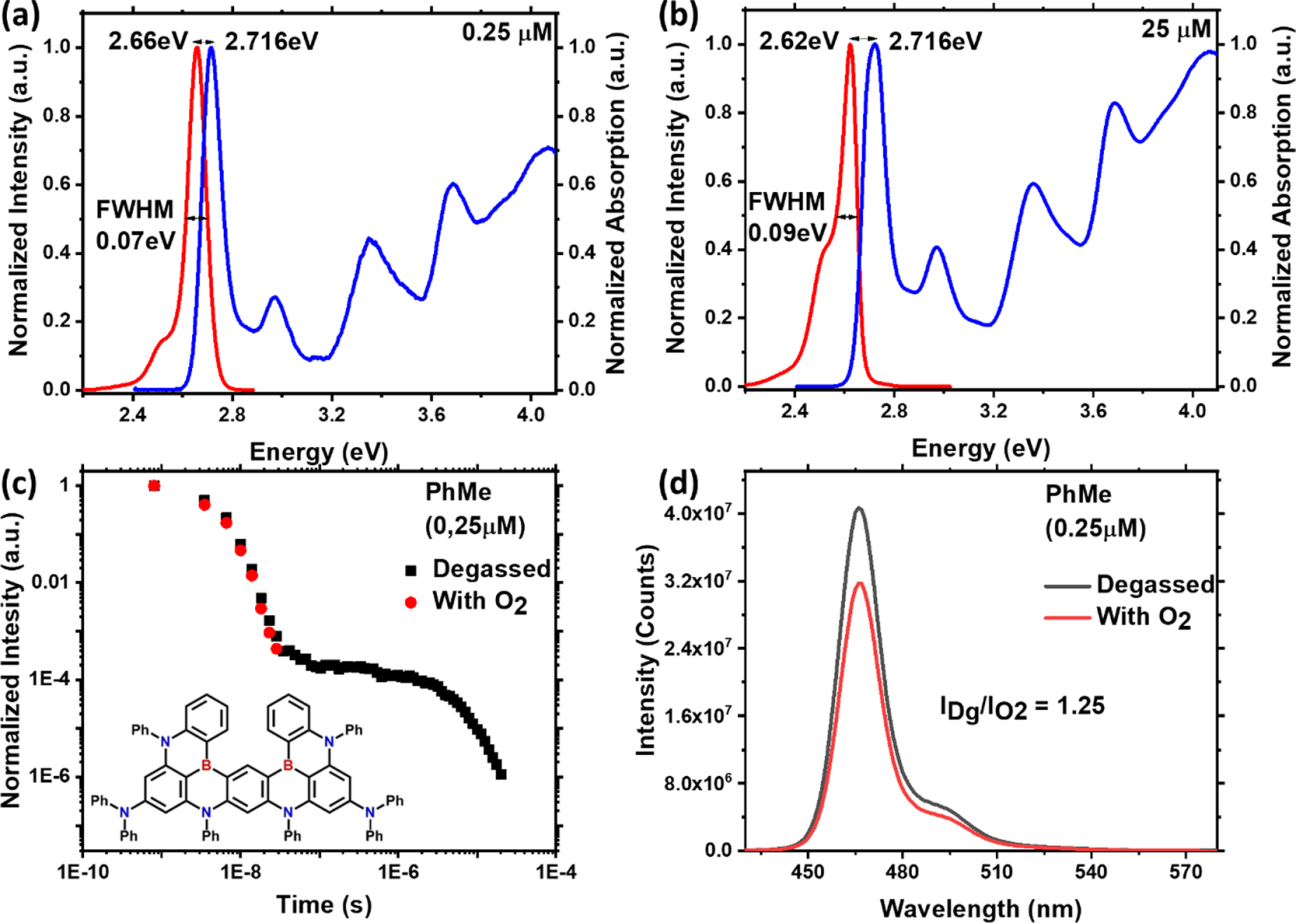
Absorption (blue) and fluorescence (red) spectra of ν-DABNA in toluene solution (a) 0.25 μM and (b) 25 μM concentration at 298 K. The effect of oxygen on prompt and delayed emission is shown in (c) (inset ν-DABNA structure) and ratio of total emission in (d).

Recently, we developed a new MR-TADF material, *N*^7^*,N*^7^*,N*^13^*,N*^13^,5,9,11,15-octaphenyl-5,9,11,15-tetrahydro-5,9,11,15-tetraaza-19b,20b-diboradinaphtho[3,2,1-de:1′,2′,3′-jk] pent-acene-7,13-diamine (ν-DABNA), with a fully resonant, extended π-framework (Figure 1).^18^ ν-DABNA showed an increased *k*_rISC_ (∼10^5^ s^–1^) and thus allowed us to fabricate an OLED with an excellent external quantum efficiency (EQE) with minimal efficiency roll-off (34.4%/26.0% at 15/1000 cd m^–2^). Moreover, ν-DABNA exhibited extremely narrower-band emission (FWHM of 14–18 nm), which is comparable to those of well-defined LEDs based on gallium nitrides and CdS/ZnS quantum dots. However, despite these notable properties, its photophysics has largely been unexplored so far. Herein, we report detailed photophysical analysis based on steady-state and time-resolved photoluminescence (PL) measurements along with hyperfluorescence (HF)-type OLEDs. We were able to elucidate the underlying mechanisms of MR-TADF in the DABNA-type materials, identifying both intrinsic and extrinsic factors that limit the current emission linewidth and rISC performance.

## Experimental Section

### Sample Preparation

Photophysical characterization of solutions was performed in five different solvents: toluene (PhMe), dichloromethane (DCM), chlorobenzene (CB), acetonitrile (MeCN), and 2-methyltetrahydrofuran (2-MeTHF). The solutions were prepared at a concentration of 25 μM, except MeCN and 2-MeTHF solutions at 0.25 μM because of solubility reasons. In PhMe and DCM solutions, for the additional concentration study, a range of concentrations between 2.5 nM and 25 μM were used. All solutions were degassed by five freeze-pump-thaw cycles. Solid-state samples were fabricated by the solution-casting technique onto quartz and transparent sapphire substrates. All host–guest films were produced in a range of concentrations between 0.0001 and 10 wt % of ν-DABNA in the host matrix (Zeonex, DPEPO (bis[2-(diphenylphosphino)phenyl]ether oxide),) and UGH (*m*-bis(triphenylsilyl)benzene)). For a neat film, a solution of 0.1 mg/mL, in toluene, was used.

### Photophysical Characterization

Steady-state absorption and emission spectra were measured using a double beam Shimadzu UV-3600 UV/VIS/NIR spectrophotometer and a Horiba Jobin Yvon Fluorolog-3 spectrofluorometer. Time-resolved measurements were performed using a spectrograph and a gated iCCD camera (Stanford Computer Optics), where samples were excited with a Nd:YAG laser (EKSPLA), 10 Hz, 355 nm or using a nitrogen laser, 10 Hz, 337 nm, for power dependence measurement.

### Device Fabrication

OLEDs were fabricated on patterned indium tin oxide (ITO)-coated glass (VisionTek Systems) with a sheet resistance of 15 Ω/sq. Oxygen-plasma cleaned substrates were loaded into a Kurt J. Lesker Super Spectros deposition chamber, and both the small molecule and cathode layers were thermally evaporated at pressure below 10^–7^ mbar. The materials used for the production of the TADF-only devices were *N*,*N*-bis(naphthalene-1-yl)-*N*,-bis(phenyl)benzidine (NPB) as the hole injection layer, 4,4′-(Diphenylsilanediyl)bis(*N*,*N*-diphenylaniline) (TSBPA) as the hole transport layer, the emissive layer (EML) had bis[2-(diphenylphosphino)phenyl]ether oxide (DPEPO) as a host and the TADF emitter, 2,2,2″-(1,3,5-Benzinetriyl)-tris(1-phenyl-1-H-benzimidazole) (TPBi) as the electron transport layer, lithium fluoride (LiF) as the electron injection layer, and aluminum (Al) cathode. The HF-type OLEDs had the same structure plus a small percentage of *N*^7^,*N*^7^,*N*^13^,*N*^13^,5,9,11,15-octaphenyl-5,9,11,15-tetrahydro-5,9,11,15-tetraaza-19b, 20b-diboradinaphtho[3,2,1-de:1′,2′,3′-jk]pentacene-7,13-diamine (ν-DABNA) as part of the EML. NPB, TPBi, and DPEPO were purchased from Sigma-Aldrich and sublimed before use. TSBPA was purchased from Lumtec and used as received. ν-DABNA was synthesized as previously reported.^18^

### Device Characterization

Freshly evaporated devices were encapsulated under an inert atmosphere using UV-curable epoxy (DELO Katiobond) along the outer edges of the active area with a glass coverslip. Devices were then transferred into a calibrated 10-inch integrating sphere (Labsphere), and their electrical properties were measured using a source meter (Keithley 2400). Emission spectra were simultaneously measured using a calibrated fiber coupled spectrometer (Ocean optics USB4000). All encapsulated devices were evaluated at RT (298 K) and under an air atmosphere.

### Crystallographic Data Collection and Structure Determination

The crystal data of ν-DABNA were collected on a Rigaku Mercury375R/M CCD (XtaLAB mini) diffractometer using curved graphite monochromated Mo Kα radiation (λ = 0.71075 Å). The reflection data for ν-DABNA were integrated, scaled, and averaged using CrystalClear. Semiempirical absorption correction was applied using the program of Abscor.^19^ The structures were solved by direct methods (SIR2014^20^) and refined by the full-matrix least squares method on *F*^2^ for all reflections (SHELXL-2014/7^21^). All hydrogen atoms were placed using AFIX instructions (C–H = 0.95 Å and O–H = 0.84 Å), while all other atoms were refined anisotropically. In the subsequent refinement, the function ∑*w*(*F*_o_^2^ – *F*_c_^2^)^2^ was minimized, where |*F*_o_| and |*F*_c_| are the observed and calculated structure factor amplitudes, respectively. The agreement indices are defined as R_1_ = ∑(||*F*_o_| – |*F*_c_||)/∑|*F*_o_| and wR_2_ = [∑*w*(*F*_o_^2^ – *F*_c_^2^)^2^/∑(*wF*_o_^4^)]^1/2^. All calculations were performed using Yadokari-XG 2009,^22^ and illustrations were drawn using ORTEP–3.

## Results

The basic photophysical properties of ν-DABNA in toluene solution are characterized by a multicomponent absorption spectrum of narrow bands, dominated by the leading contribution, Figure 1. The progression is however not a vibronic progression as there is not a consistent energy separation between each band and so we ascribe these bands to higher energy electronic transitions, that is, S_1_, S_2_, S_3_...., with S_2_ being only 250 meV above S_1_, Figure S1. The S_1_ band is on-set at 2.66 eV, with peak absorption at 2.72 eV and a FWHM of 70 meV (ν-DABNA 0.25 μM in toluene). The absorption is mirrored by a sharp emission band, on-set at 2.75 eV, and a peak at 2.66 eV. At higher concentration (25 μM in toluene), the emission band red shifts by 40 meV and broadens to 100 meV FWHM, primarily because of a shoulder on the red edge of the main emission peak, Figure 1 and Figure S2. From time-resolved emission measurements, Figure 1c, we observe a long-lived delayed fluorescence contribution to the overall emission, which is completely quenched by oxygen, Ivac/Iox = 1.25, Figure 1d.

These spectroscopic observations are consistent with the rigid π-conjugated, near planar molecular framework of ν-DABNA,^18^ dominated by absorption and emission from the same lowest energy electronic transition (resulting in the symmetric absorption/emission profiles). We take the true band shape of the narrow S_0_ ← S_1_ transition to be represented by that observed in 2.5 nM toluene solution, Figure S2, indicating a strong leading component and a weak vibronic side band, intensity ratio 1:0.12, and energy separation of ca. 120 meV (960 cm^–1^), Table S2. This indicated that a vibrational mode strongly couples to the S_1_ electronic transition. This analysis also confirms that higher energy absorption bands are therefore not vibronic replicas of the lowest excited state. Excitation profiles (of film samples) reveal a finer vibronic structure on the lowest energy absorption band, Figure S3. Fitting of these features gives a consistent vibronic progression with an energy spacing of ca. 285 cm^–1^, Table S1, consistent with coupling to stretching/scissoring modes of the peripheral phenylene rings.^18^ The arrangement of excitonic states gives rise to both the small Stokes shift (14 nm, 450 cm^–1^) and FWHM (70 meV), in zeonex at low concentration, of the S_0_ ← S_1_ emission band.

The emission and absorption spectra are only weakly affected by solvent polarity, Figure S4. This lack of solvatochromic shift and no obvious change in the line shape as solvent polarity increases indicate no apparent charge transfer (CT) character to the first excited state. The peak extinction coefficient at 3 × 10^5^ M^–1^ cm^–1^ (250 nM, toluene), Figure S2c, and a minimal Stokes shift (14 nm) indicate an excitonic nature to this lowest energy excited state.

In toluene, the main emission band is observed to start to red shift at concentrations above 2.5 μM, which we ascribed mainly because of self-absorption and the inner filter effect (we see no red shift of the absorption band on the red side of the band), Figure S2f. From 2.5 nM to 2.5 μM, the long wavelength side of the emission band also starts to increase in intensity, indicating the emergence of a new emission band. As a result, the emission spectrum FWHM also increases. The same phenomenon is observed in higher polarity DCM, Figure S2f, with a slightly smaller redshift of the main emission peak and a smaller contribution from the new low energy band, Table S3. We thus identify this as a new band, underlying the exitonic emission band that increases with concentration and shifts position with the environment.

Molecular rigidity and planarity usually enhance intermolecular interactions such as π-stacking and dimerization. While the peripheral phenyl rings of ν-DABNA are sterically orthogonal to the molecular core, which should hinder such interactions, recent reports have shown that phenyl substituents on large aromatic structures can still suffer significant flattening outside of solution or gas phase.^23^ The increased intensity on the red side of the emission band, which is seen in all solvents as well as in films and with the position of the underlying feature found to be dependent on the environment, is therefore characteristic of an aggregate or excimer state. The ratio between the main S_0_ ← S_1_ transition and the underlying band is found to increase nonlinearly with concentration in solution, Figure S5. We find no evidence of a weak absorption band below the excitonic absorption band that might otherwise be associated with a ground state dimer. Thus, we conclude that this red emission comes from an excimer state.

**Figure 2:**
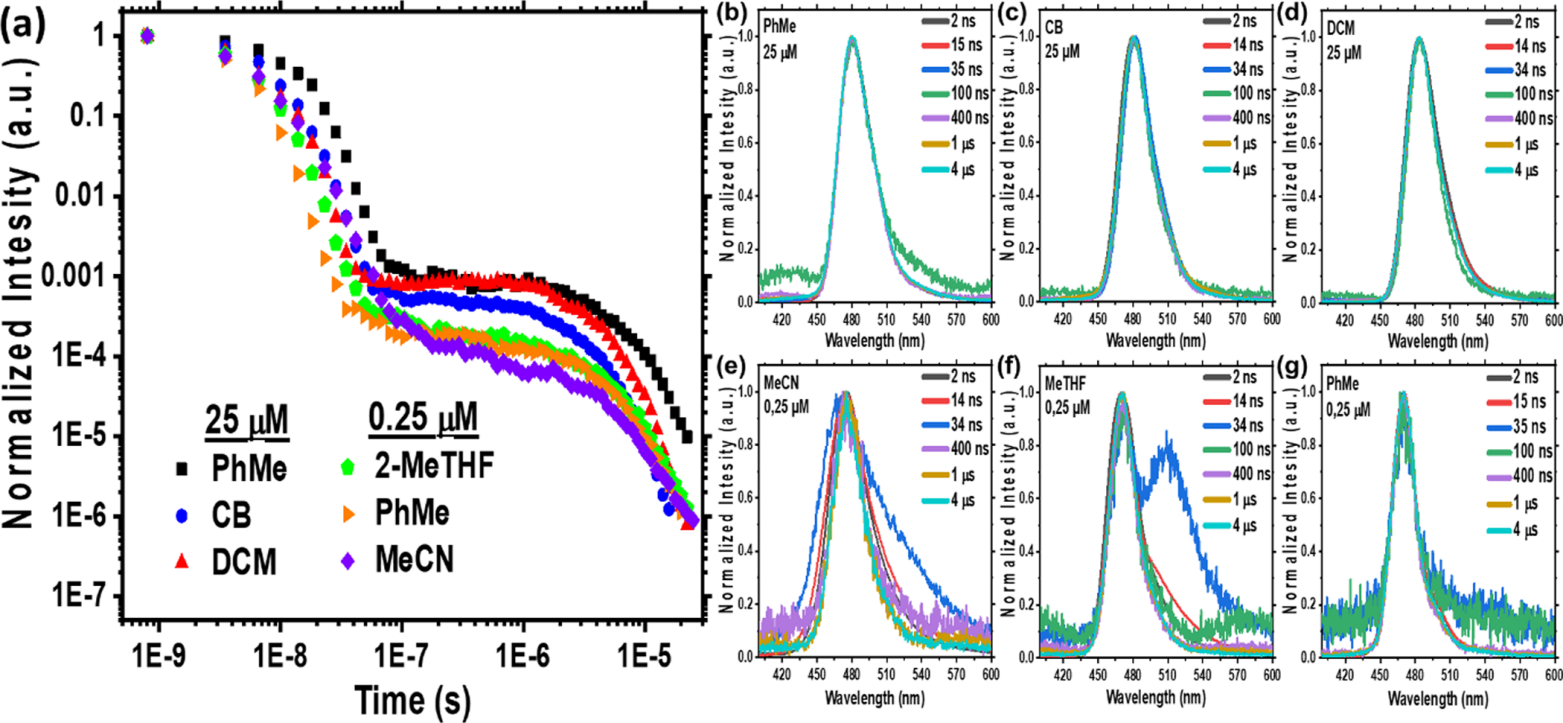
Time-resolved PL spectra of ν-DABNA in (b) PhMe solution 25 μM, (c) CB solution 25 μM, (d) DCM solution 25 μM, (e) MeCN solution 0.25 μM, (f) 2-MeTHF solution 0.25 μM, and (g) PhMe solution 0.25 μM concentration, at 298 K. (a) Time-resolved PL decays of all the previous. Excitation at 355 nm.

Time-resolved transient PL measurements of ν-DABNA in a range of solvents, with different dielectric coefficients from 2.21 to 37.5 and viscosities (Table S4), are shown in Figure 2. The environmental polarity affects the intermolecular excimer interactions, with the red component emission becoming more intense in some solvents, most notably 2-MeTHF. For two concentrations of ν-DABNA in toluene; 0.25 μM (prompt lifetimes: 3.4 ns and 30.7 ns; delayed lifetime: 3.6 μs) and 25 μM (prompt lifetimes: 10 and 54 ns; delayed lifetime: 5 μs), Figure S6, it is also apparent that increasing concentration extends the lifetimes of the prompt and delayed emission. The longer of the two prompt lifetimes we therefore ascribe to the excimer state.

The decay kinetics of the emission of ν-DABNA was measured in the same range of solvents. The decays were analyzed following the kinetic fitting model previously reported by Haase et al.,^24^Figure S7. From this, we find that the reverse intersystem crossing rate (krISC) is, within error, independent of the solvent polarity, ranging only between 3 × 10^5^s^–1^ and 5.5 × 10^5^s^–1^. There is also no correlation of krISC with solvent viscosity, Table S4, also indicating that the mechanism of DF in ν-DABNA is analogous as in intramolecular CT, D-A type, molecules.^25,26^ We find that the fitted intersystem crossing (ISC) rate is an order of magnitude less than the radiative decay rate, consistent with a measured PLQY of 0.7–0.9.^18^ This indicates that internal conversion in ν-DABNA must be very low, as expected for a highly rigid molecule.

**Figure 3:**
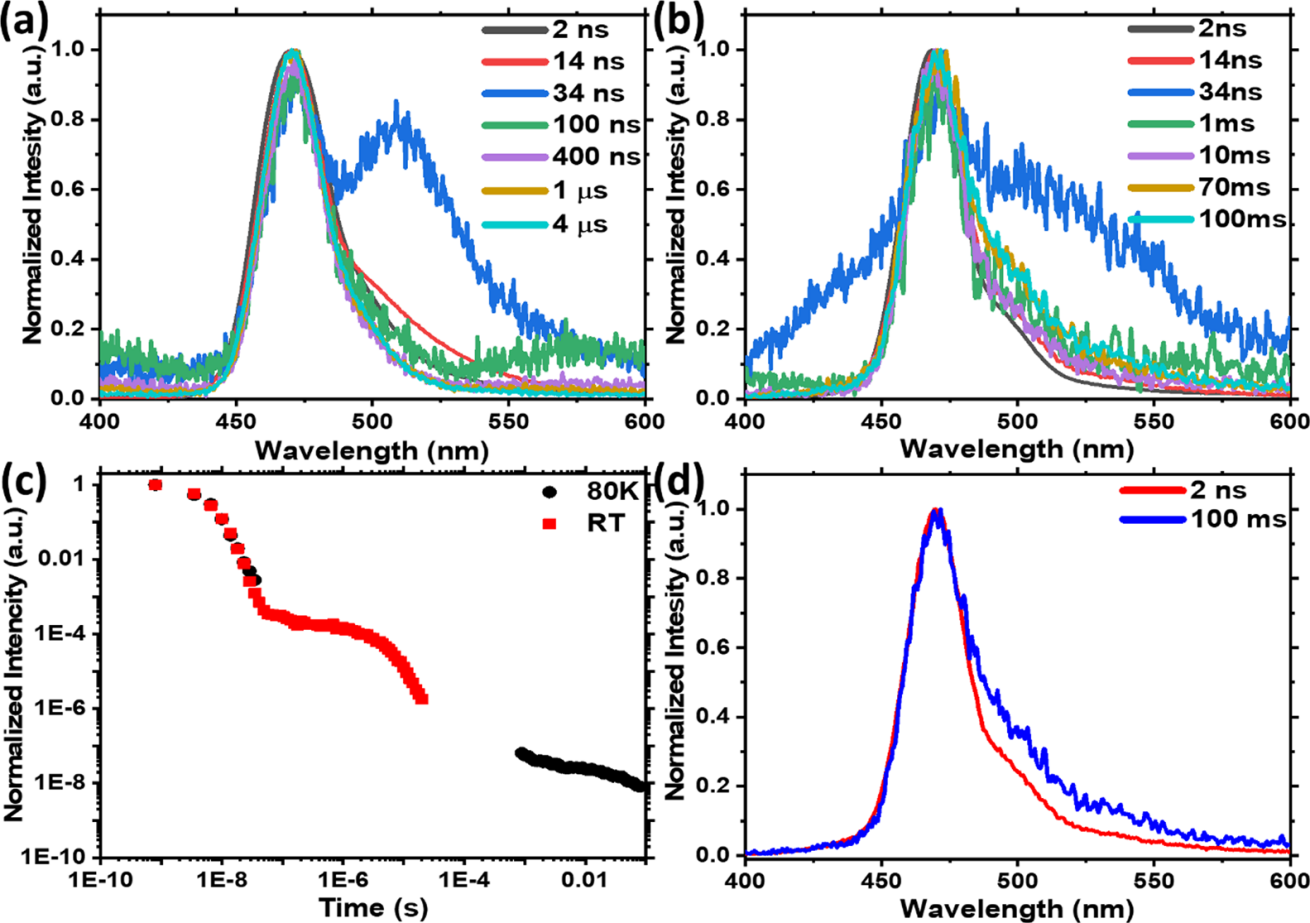
Prompt and delayed time-resolved PL spectra of ν-DABNA in 2Me-THF solvent, 0.25 μM concentration, at (a) RT and (b) 80 K. (c) Time-resolved PL decay at both temperatures and (d) prompt and phosphorescence emission at 80 K. Excitation at 355 nm. Breaks in the decay curve indicate a signal below the noise floor of our detection system, indicating that extreme or no sample signal is present.

As the excimer state is most readily observed in the time-resolved spectra of 2Me-TFH solutions, we also investigated this glass-forming solvent at cryogenic temperatures. At low concentration (0.25 μM) and RT, Figure 3a, the initially excited exciton state decays with a lifetime of 4.5 ns. A second lower energy emission band grows in over 50 ns (multiexponential fittings in Figure S8). This band decays within 100 ns to leave a long-lived delayed emission band, which persists into the 10s of μs. The DF clearly arises only from the exciton emission, with no DF arising from the transient red species. Lower temperatures (80 K) completely suppress the rISC mechanism, and no DF is observed, Figure 3c. Comparing RT to 80 K, at early times (2 ns) the emission is dominated by the intense exciton band. At 80 K, the linewidth of the excimer band increases dramatically, with its on-set increasing to 3.10 from 2.77 eV, thus extending to energies higher than the exciton emission, Figure 3b. Such a broad emission spectrum indicates a large distribution of inhomogeneous excimer states/geometries with emission energy highly dependent on the distance and orientation between the interacting molecules.

In addition to the excimer emission, at low temperatures a weak but very long-lived emission is observed, with an identical spectrum to the exciton S_0_ ← S_1_ transition, Figure 3d. This is indicative of local triplet state phosphorescence that is degenerated with the singlet exciton and so Δ*E*_ST_ is close to zero. Such degeneracy implies that spin orbit coupling (SOC) between these two states is forbidden^27^ and that some other thermally activated mechanism must give rise to the strong TADF at RT but which is totally suppressed at 80 K. Again, no DF or phosphorescence arises from the excimer state.

**Figure 4:**
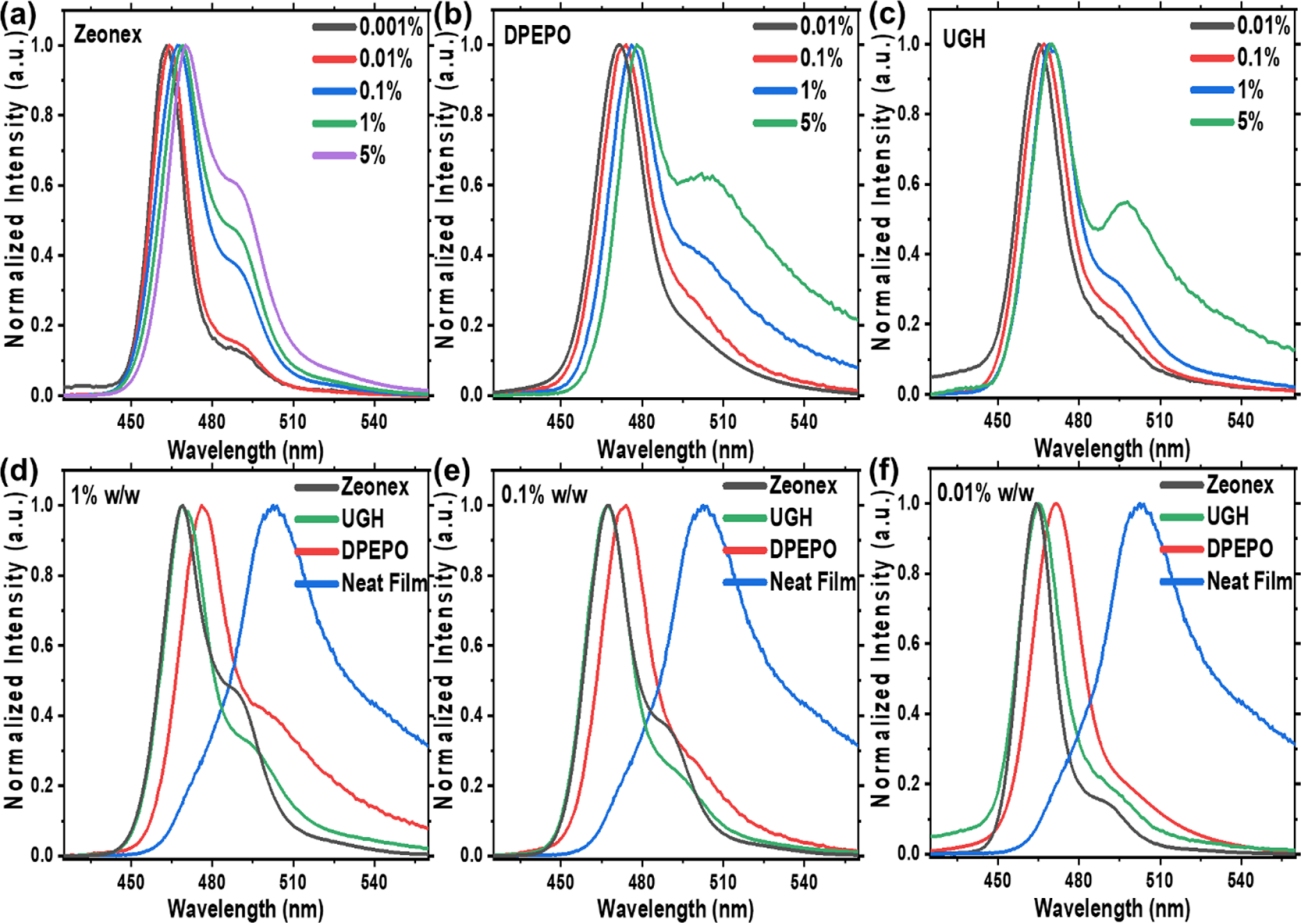
Normalized RT PL spectra of ν-DABNA in (a) zeonex, (b) DPEPO, and (c) UGH matrix, in various concentrations. Comparison of different hosts and neat film in (d) 1 wt %, (e) 0.1 wt %, and (f) 0.01 wt % concentration of emitter to host ratio. Excitation at 370 nm.

Solid-state measurements greatly add to this complex picture. A set of different host materials were investigated; cyclic olefin polymer (Zeonex), a nonpolar soft polymer matrix; *m*-bis(triphenylsilyl)benzene (UGH), a low dielectric rigid matrix; and bis[2-(diphenylphosphino)phenyl] ether oxide (DPEPO), a high dielectric and rigid matrix. Neat films of ν-DABNA were also investigated in which excimer formation was anticipated to be maximized. Steady-state emission spectra at different ν-DABNA loadings are given in Figure 4.

Zeonex gives a certain degree of freedom to the motion of the molecule, but less than a solution. Thus, it acts as an intermediate between solution and solid-state materials used as OLED hosts, facilitating comparison and in-depth investigation of the excimer contribution and concentration dependence in each. We observe a clear onset of excimer emission contribution above 0.01 wt % loading in zeonex, Figure 4a. As was seen in solution, these data indicate that the increasing excimer emission is intermolecular in nature, and no change in ground state absorption is seen. In both UGH and DPEPO, we also see increasing emission from the excimer state as concentration increases. The excimer emission starts at very low loading levels, well below those used in device applications, indicating a very strong association constant for the ν-DABNA excimer. In the neat film, the excimer emission band totally dominates, as might be expected. The neat film spectra show some exciton emission (as a knee on the blue edge of the emission) as well as an indication of the highly disordered solid, with a distribution of excimer geometries and energies resulting in a long excimer band tail stretching past 575 nm. In UGH and the DPEPO matrix (5 wt % films), the peak of the excimer band corresponds very well with the peak of the neat film emission, supporting our interpretation of shared excimer formation in each.

A more detailed set of concentration measurements were made in zeonex, ranging from 1 × 10^–4^ to 1 wt % loading of ν-DABNA in zeonex. The two emission bands observed were fitted using Voigt band shapes and the relative change in intensity of the two transitions plotted, Figure S9. We observe that the excimer band intensity increases faster than the increase of the exciton band intensity as concentration increases. Similar behavior is seen in solution but only at the highest concentrations and may indicate exciton migration and/or energy transfer to excimer-forming sites in film. This also shows that excimers are stabilized in the solid-state, compared to solution where they can more freely diffuse and dissociate. The maximum relative PL intensity of the exciton band is found between 0.01 and 0.1 wt % ν-DABNA, with reductions even at 0.01 wt % loading. Furthermore, the exciton and excimer transitions redshift comparably with increased concentration in zeonex, Figure 4a and Table S5. In UGH and the DPEPO matrix, the excimer band red shifts further compared to the exciton emission, Figure 4, indicating that the (energy) distribution of excimer states changes with concentration in rigid environments (Tables S6 and S7).

**Figure 5:**
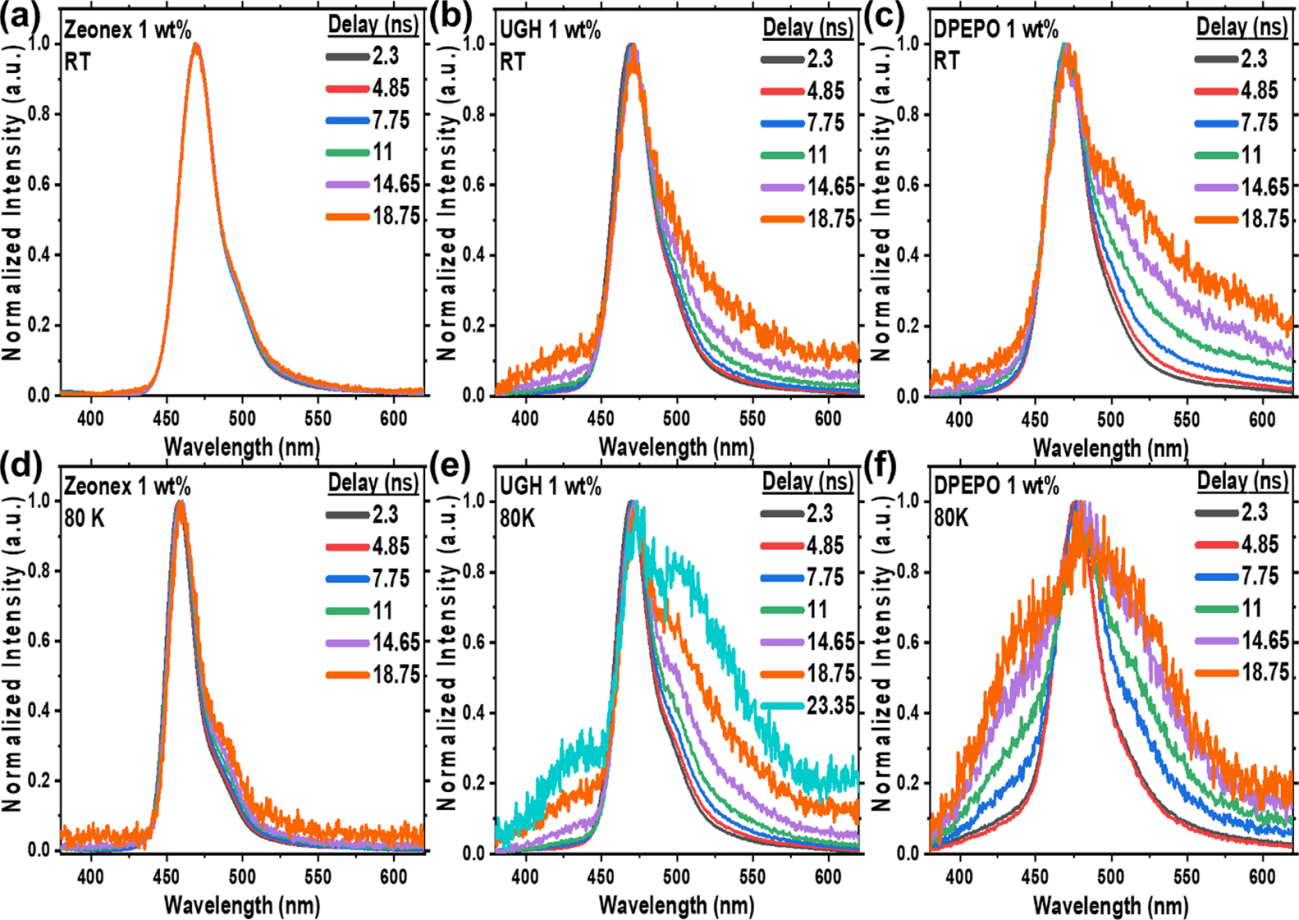
Time-resolved prompt emission of ν-DABNA, 1 wt %, in zeonex matrix (a) RT, (d) 80 K, UGH matrix (b) RT, (e) 80 K and DPEPO matrix (c) RT, and (f) 80 K. Excitation at 355 nm.

The temporal evolution of the excited states was again followed in the early nanosecond time regime, Figure 5. At 1 wt % loading in zeonex, there is no clear excimer contribution at RT and only a very small signature at 80 K, in contrast with 0.01 wt % loading where only excitonic emission is observed, Figure S10. In UGH (1 wt %), we again observe initial excitonic emission with the grow-in of the excimer band over 20 ns. From the full kinetics trace, Figure S11a, we observe characteristic prompt emission from the exciton that decays with a lifetime of 3.5 ns and a very broad excimer band with a lifetime of 50 ns. DF is observed only from the excitonic band with a lifetime of 2.5 μs. Very similar lifetimes were also found in the DPEPO host with the DF having a lifetime of 3.2 μs, Figure S11b. The excimer band can be observed for more than 100 ns, having a Gaussian line shape and an extremely large FWHM. We suggest that this long lifetime arises from the rigidity of ν-DABNA molecules and extremely weak vibrational coupling, meaning that nonradiative decay does not interfere despite slow rISC. Laser excitation intensity measurements confirmed that the DF in UGH is linear with excitation power, excluding a bimolecular DF mechanism, Figure S12. This rules out the TTA mechanism for DF generation at RT in ν-DABNA.^28^

**Figure 6:**
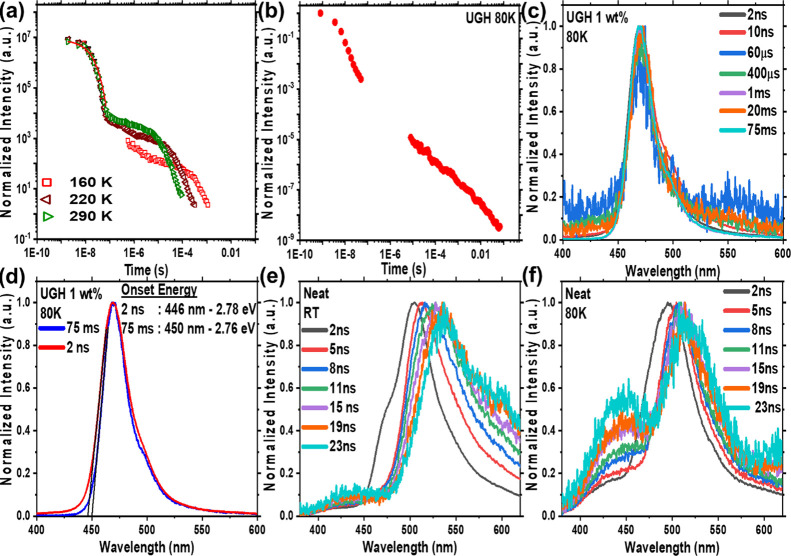
Time-resolved, (a) temperature-dependent DF decay kinetics, (b) decay at 80 K and (c) spectra, at 80 K, with longer integration times, revealing an extremely weak long-lived decay component measurable from 10 μs into the ms where it merges with phosphorescence, and (d) singlet-triplet gap of ν-DABNA 1 wt %, in the UGH matrix; evolution of the PL emission for the ν-DABNA neat film at (e) RT and (f) 80 K. Excitation at 355 nm.

As the temperature is lowered, we see an evolution of the emission decay curves, Figure 6a, with DF being less intense but with increasing lifetime. At 80 K, Figure 6b, the DF is very weak, and the decay is no longer exponential. Long exposure measurements reveal that the emission from 10 μs to the 75 ms does not change and is still perfectly representative of the exciton emission seen in the ns, Figure 6c. This could indicate that once the monomolecular triplet spin upconversion mechanism is frozen out, weak TTA can still occur, consistent with a triplet population no longer being depleted by the upconversion process. At 80 K, the excimer band behaves as it does in frozen 2Me-THF, being very broad with on-set at higher energy than the excitonic emission, Figure 5e and Figure S15.

In DPEPO, the temporal evolution of the excimer emission is much faster. At RT, we see strong quenching of the exciton band by 20 ns and the grow-in of a well-defined excimer band (at the same spectral position as neat film emission). Similar to UGH, by 20 ns, the total emission intensity is already very weak. However, at 80 K, the behavior is distinct from UGH or 2Me-THF. We observe the well-resolved excimer band along with the near-complete quenching of the exciton emission, Figure S13. Given that we observe the emission onset and peak positions from the 0.1 wt % DPEPO film are very similar to those observed in a 1 wt % UGH film, this shows that the excimer state is not affected by host dielectric or polarizability but instead is more dependent on (different) host packing forces.^29^ As in solution, the DF from films always comes from the exciton state not the excimer, Figure S14. In all hosts, DF is highly suppressed at 80 K in line with solution measurements and again indicating that a different rISC mechanism is at work in ν-DABNA compared to intramolecular D-A TADF molecules.

The time-resolved emission from neat films of ν-DABNA reveals more precisely what we are seeing in the different guest–host films, Figure 6e,f. At 80 K, everything is dominated by the new species, and any sharp 460 nm exciton emission is weak or masked by other emission. Instead we observed a fast decaying emission around 440 nm. This is broad with a Gaussian line shape and a lifetime of 5.5 ± 1.5 ns, Figure S16. The second band is at lower energy, peaking at 510 nm with a distorted Gaussian-like band shape and a lifetime of 4.2 ± 2.6 ns. From these spectra, as well as those observed in UGH and DPEPO, these are two distinct spectral bands corresponding to different emitting species, which are highly dependent on temperature and concentration. Total emission in the neat films is, however, very weak, likely suffering strong aggregation quenching and with errors the lifetimes such that they might be equivalent. At RT, clearly a high-energy band is not observed, although we do observe the exciton band at 460 nm that is quenched within 5 ns along with the lower energy emission band. This is very similar to the feature observed in 10 wt % ν-DABNA in DPEPO, Figure S13, and dominates the neat film steady-state emission, Figure 4. At RT, the red species clearly red shifts in time, indicating possible exciton migration to lower energy sites, leading to rapid excited state quenching within the film. At 80 K, this is not observed. Because of this, we never observe delayed emission in the neat film and conclude that exciton migration significantly impacts the properties of the neat film.

Further considering the neat films, at RT the high-energy state is absent, whereas at 80 K, we observe emission from both bands. This clearly suggests that these are two distinct excimer states, pointing to two different orientations of ν-DABNA molecules giving two different excimer configurations. The high-energy state must be thermally unstable, that is, weakly bound, so it is not observed at RT nor do we see it in zeonex at 80 K where the ν-DABNA molecules have more free volume to reorient. These spectral features are dominant in the neat film and are observed for high-concentration guest–host films and frozen 2Me-THF glasses, all consistent with excimer states. However, we also see a lower energy emission band in many environments at RT. In 2Me-THF, it grows in within 30 ns and decays completely by 100 ns, as compared to films where it is observed from the first time frames. Further in frozen 2-MeTHF and UGH at 80 K, Figure 5, everything becomes extremely broad and ill-defined, indicative of large scale disorder. All of these observations taken together indicate excimer states that are weakly coupled and highly disordered in films especially at low temperatures, with additional excimer geometries becoming prevalent at higher concentrations.

Detailed analysis of the time-resolved emission decay in zeonex at the optimum concentration (0.01 wt %) to avoid excimer states yields lifetime and kinetic results similar to those in solution, Figures S10 and S17. The singlet-triplet gap at 80 K measured using the 14 ns and 75 ms spectra Figure S10e is effectively zero, giving fast ISC even at low temperatures, but again the rISC rate was found to be 2.9 × 10^5^ s^–1^ as with all solutions and independent of the singlet-triplet gap. No DF was measurable at 80 K, Figure S10a. In UGH and DPEPO, we estimated a rISC rate of ca 6 × 10^5^ s^–1^, Figure S17. The rISC rate again appears totally independent of the molecular environment.

**Figure 7:**
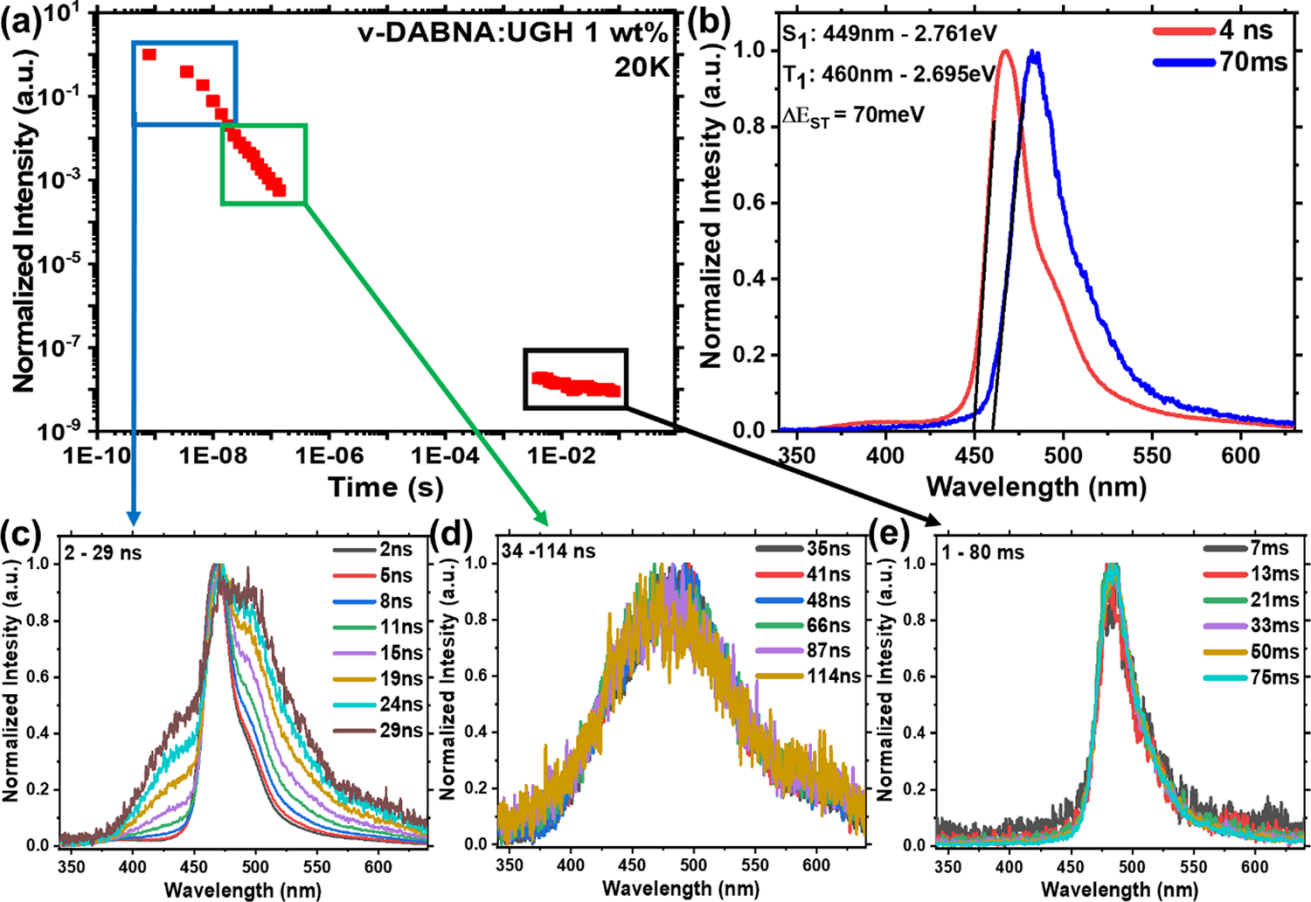
Time-resolved (a) PL decay curve, (b) singlet-triplet energy gap and PL spectra at a time range of (c) 0–30 ns and (d) 35–120 ns, and (e) phosphorescence at 10–75 ms, of ν-DABNA 1 wt % in the UGH matrix at 20 K. Excitation at 355 nm.

From the frozen 2Me-THF and zeonex film measurements, we estimate that the singlet-triplet gap (Δ*E*_ST_) is very small, of order <5 meV. To investigate the nature of the Δ*E*_ST_ further, we measured ν-DABNA UGH films in more detail. Starting from the low-temperature measurement at 80 K, the Δ*E*_ST_ gap was estimated from the difference in band on-set energies (2 ns and 75 ms spectra) to be 20 ± 5 meV, Figure 6d. However even at 75 ms, the phosphorescent emission had not stabilize at a certain energy, that is, it continued to red shift with increasing time until it was undetectable. Thus, we made a set of measurements at 20 K, Figure 7, where vibrational motion is minimized and rISC completely eliminated, so that the phosphorescence at 20 K is observed free from any DF. Even in the 20 K measurement, the phosphorescence spectrum (75 ms) is nearly identical to the singlet exciton emission showing that it has an identical orbital character to the singlet, Figure 7b. This measurement indicates a triplet energy of 2.58 ± 0.05 eV (peak), 2.69 ± 0.05 eV (on-set), which stabilizes after 3 ms and remains constant until some hundreds ms, Figure 7e. This yields a Δ*E*_ST_ of 70 meV (from the band on-sets), which is in excellent agreement with the singlet-triplet gap estimated from Arrhenius analysis previously.^18^ Thus, we clarify that this is the energy of the lowest triplet state of ν-DABNA but conclude that the triplet energy is temperature-dependent, so at 80 K it increases by some 50 meV. We speculate that this could be through a small decrease in the conjugation of the lowest triplet state at high temperature. TD DFT calculations previously showed small torsional changes of the peripheral phenyl rings in the excited state, especially those in the tertiary amine groups, which could account for this.^18^

One very intriguing observation for ν-DABNA in UGH (1 wt %) is that the FWHM of the exciton emission band (5 ns) increases as the temperature increases, from 109 meV at 20 K, to 137 meV at 80 K, and 157 meV at RT. We also observe that the shape of the early time RT exciton band is poorly defined, unlike at lower temperature. Furthermore, a 65 meV red shift of the energy of the exciton band is seen between RT and 20 K, which arises through a loss of the blue edge emission at low temperatures. Very similar behavior is also seen for DPEPO 1 wt % films. This indicates a large population of vibrational excited states at RT contributing to radiative decay, which become deactivated at lower temperatures. Given that we have shown the coupling to a 35 meV (285 cm^–1^, Figure S1) vibrational mode to this exciton transition, at RT the populations of the first two excited vibronic sublevels of S_1_ will have very high Boltzmann factors of 0.5 and 0.25, respectively. This then gives rise to ‘hot transitions’ from these populated states (S_1,1_ and S_1,2_) to S_0,0_ pushing the band on-set further into the blue compared to that at cryogenic temperatures.

From the time-resolved spectra measured at 80 K in zeonex 0.01 wt %, Figure S10 and DPEPO 10 wt %, Figure S15, we observe a red shift of the (whole) prompt exciton emission band over the first 20 ns, whereas at RT, there is little shift. The maximum shift is ca. 36 meV (290 cm^–1^) over 8 ns. This corresponds very well with the energy difference calculated between the S_1_@S_0_ and S_1_@S_1_ geometries and from the decomposition of the absorbance spectrum,^18^ which could indicate a small planarization of the ν-DABNA molecule over this time at low temperatures. However, we see no shift at RT, 80 or 20 K in the UGH host, Figure 5 and Figure 7. This may indicate that UGH packs more tightly than both zeonex and DPEPO constraining ν-DABNA molecules in the ground state configuration, while these vibrations are continually re-equilibrated through the decay leading to no apparent wavelength shift.^29^ Thus, we can observe effects of both molecular vibrational motions of the core and peripheral units of the ν-DABNA on the FWHM and emission band position, which give rise to appreciable increases to the FWHM at RT.

**Figure 8:**
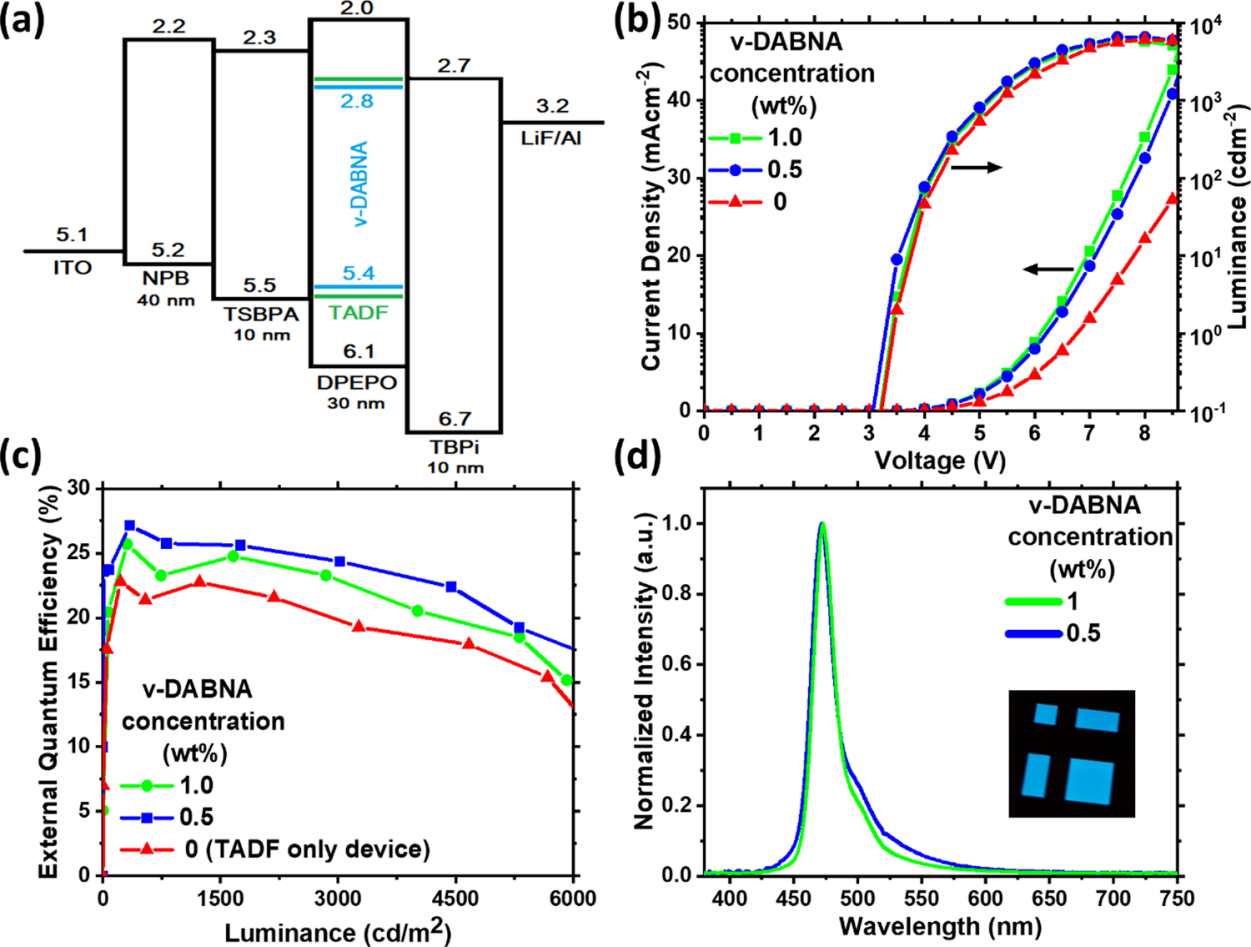
OLED performance for TADF only (red) and HF 0.5 wt % (blue) and 1 wt % (green) concentration of ν-DABNA. (a) Device structure and ionization potentials and electron affinities (in eV) for each layer. (b) Current density and luminance vs. driving voltage. (c) EQE vs. luminance. (d) Normalized EL spectra. Inset: Operating device*.*

To evaluate the effect of excimer state electroluminescence (EL) performance, devices using ν-DABNA as a HF-type emitter were fabricated and analyzed. This approach has recently emerged as a strategy to achieve excellent device color from the ν-DABNA, with improved rISC and roll-off characteristics provided by the TADF cohost.^15,30−33^ The devices had the following structure: ITO; *N*,*N*-bis(naphthalene-1-yl)-*N*,-bis(phenyl)benzidine (NPB, 40 nm); 4,4′-(Diphenylsilanediyl)bis(*N*,*N*-diphenylaniline) (TSBPA, 10 nm); 65-x wt % DPEPO, 35 wt % TADF emitter, and x wt % ν-DABNA HF emitter (30 nm); 2,2,2″-(1,3,5-Benzinetriyl)-tris(1-phenyl-1-H-benzimidazole) (TPBi, 10 nm); lithium fluoride (LiF, 0.8 nm); aluminum (Al, 100 nm). In previous work, the optimum ν-DABNA emitter concentration was estimated to be 1 wt %.^18^ To explore the excimer effect in this work, we compared 1 to 0.5 wt % doping, approaching the lower limit of doping achievable by triple coevaporation. The V-I-L characteristics and HOMO/LUMO levels of the materials are shown in Figure 8 and compared to those of the control TADF device containing no ν-DABNA. The cyan D-A-D TADF emitter employed as a cohost is based on a simple modification of a previously reported series of TADF molecules, Figure S19.^34^ By introducing ν-DABNA into the device, the EQE increased by 13.3% for 1 wt % and 22.2% for 0.5 wt % concentration (at approximately 4.5 V, 1 mAcm^–2^ and 850 cdm^–2^ in both cases), compared to the control device, Figure 8c. The emission color was also significantly improved in the ν-DABNA devices, Figure 8d, because of the narrow emission band compared to the CT emission of the D-A-D cohost, Figure S19.

As anticipated, the use of ν-DABNA with a previously reported maximum EQE of 34.4% using an optimum DOBNA-Oar host^18^ leads to a major increase in device efficiency. However, given that some of this performance has since been demonstrated to arise from spontaneous molecular alignment of the ν-DABNA and nonlambertian angular emission profile leading to enhanced device outcoupling,^30^ the performance of our HF devices is still not as high as expected. In the best-case scenario, an estimated 1.3-fold outcoupling enhancement because of aligned emission combined with the very high intrinsic EQE and rISC of the TADF cohost in the control device would lead to EQEs >30% in our hyperfluorescent devices. This comparison leads to the conclusion that a variety of mechanisms must suppress the performance below what may otherwise be achievable. According to our photophysical studies, the excimer state quenches emission even at concentrations close to 0.1 wt %. Thus, we identify ν-DABNA excimer quenching as at least one of the causes of below-expectation EQE in these devices. Excimer quenching also explains the reduced performance as the ν-DABNA doping concentration increases from 0.5 to 1%.

The operation of HF-type OLEDs relies on Förster resonance energy transfer (FRET) from the higher singlet (S_1_) energy TADF donor to the slightly lower but much narrower FWHM S_1_ state of the HF emitter. To investigate this further, for the two different concentrations eq 1 was used to estimate the Förster radius (*R*_0_):
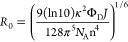
1where κ is a dipole–dipole orientation factor, here taken as 2/3 for a randomly oriented system, Φ_D_ is the PLQY of the donor in the absence of the acceptor (0.85), n is the refractive index of the host (1.671), *N*_A_ is the Avogadro constant, and *J* is the spectral overlap integral extracted by eq 2:
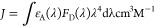
2where ε_A_ is the extinction coefficient of the acceptor, *F*_D_ is the normalized emission of the donor, and λ is the wavelength. The spectral overlap using the normalized emission spectrum of the donor (TADF emitter) and the extinction coefficient of the acceptor (ν-DABNA) are shown in Figure S20. The calculated *J* value was 1.944 × 10^15^ M^–1^ cm^–1^ nm^4^, resulting in an *R*_0_ of 4.81 nm. This is a relatively large Förster radius, but in keeping with the near-complete overlap of emission and absorption spectra in this case.

The EL spectra of control and HF devices, with 1 and 0.5 wt % doping, along with the ν-DABNA PL spectrum are shown in Figure S20b. Comparing the photoexcited emission of ν-DABNA (red) with the two different concentration HF devices, one can observe the ideal overlap at 1 wt %, translating to near-complete Förster energy transfer and a D-A distance (*r*) smaller than 0.5*R*_0_ (*r* < 2.4 nm), Figure S21. On the other hand, the 0.5 wt % HF device shows an increased emission width, especially at 0.1 maximum height, which contributes to the overall emission both at higher and lower energies. This emission we suggest comes from the TADF emitter itself, indicating incomplete FRET. As a result, Förster efficiency is lower, although evidently still above 50% because of the small contribution of the TADF emitter to the overall device emission. In this case, according to Figure S21, we estimate the D–A distance in the range of 2.4 < *r* < 4.8 nm, which is larger because of the smaller concentration of ν-DABNA.

## Discussion

From these measurements, it is clear that there are both intrinsic and extrinsic factors that control both the emission linewidth and rISC mechanism in ν-DABNA. Across the range of environments, we observe a very similar feature with an ill-defined Gaussian-like band shape peaking around 495 nm to 510 nm, which we can ascribe to an excimer band. At low temperatures, this excimer band broadens significantly, indicating molecular disorder consistent with a weakly coupled intermolecular excimer state. The steric hindrance of the peripheral phenyl rings on ν-DABNA makes close intermolecular interaction difficult, adding to the disorder and weak coupling such that the energy shift between the exciton band and the excimer is small. Nonetheless, the X-ray crystal structure of ν-DABNA, Figure S18, demonstrates the close approach of molecules below the Van der Waals radii, indicating that weak excimer interactions are indeed possible. In zeonex and solvents such as toluene, we observe much less of the excimer state, in part because such weakly interacting states also dissociate in fluid environments. However, we observe that the extremely planar, π rich ν-DABNA molecules can readily form intermolecular interactions even at exceptionally low concentrations.

The excitonic state displays minimal solvatochromism and little change in the rISC rate with solvent polarity, clearly indicating that there is little or no change in the ground and excited state dipole moment on excitation and that the excited state has a small dipole moment.^35^ This behavior contrasts strongly to D-A(-D) TADF molecules, which have large changes in between ground and excited state dipole moments.^36^ From previously reported electron density distributions in the HOMO and LUMO orbitals of ν-DABNA,^18^ there is only a small shift in electron density on excitation, similar to BODIPY fluorophores. As in BODIPY molecules, this gives a net change in dipole moment along the short axis of the molecule, whereas the transition dipole moment (TDM) would be expected to lie along the long axis of the molecule (having the largest π electron conjugation). A TDM orthogonal to the (solvent induced) change in dipole moment therefore results in negligible solvatochromism.^37,38^ The TDM aligned with the long axis of the ν-DABNA molecule would also account for the highly anisotropic emission in films and enhanced outcoupling in OLED devices observed.^30^

From the fitted decay kinetics, relatively high ISC rates indicate that SOC cannot be responsible for coupling the near-degenerate S_1_ and T_1_ states (with near-identical spectra). It is not in line with a pseudo spin-forbidden transition. All the experimental data points to this being forbidden because of orbital symmetry, that is, no change in orbital angular moment can occur during the spin flip transition. Instead this points to an efficient upper state crossing between S_1_ and energetically adjacent T_2_ or higher triplet states, within 70–100 meV. As we have shown, at RT there is a large occupancy of upper vibrational levels of S_0_. Similar hot exciton populations in S_1_ could facilitate the ISC to an upper triplet level, for example, T_2_ or T_3_. In this case, cooling the material will greatly reduce ISC as well as any concomitant rISC, as both are effectively thermally activated. This could be why we observe such a strong overall temperature dependence in the DF - all of which strongly supports both ISC and rISC transition occurring via upper triplet states. This also explains why in films where the apparent ΔE_ST_ is zero we still measure the same moderate rISC rate, with 0 to 70 meV S_1_-T_1_ energy gaps and also the same as found in solution.

As rISC in ν-DABNA is strongly thermally activated yet insensitive to the environment, with rISC rates always found to be around 3-5 × 10^5^ s^–1^ at RT in solution and film, one potential mechanism is for T_1_ states to undergo thermally activated reverse internal conversion (rIC) to an upper triplet state (T_2_ or T_3_ for example^28^) which then enables rISC back to S_1_ (or S_N_ as they are close lying, Figure S1). This mechanism was recently reported in another highly rigid TADF molecule, triquinolonobenzene, where intramolecular proton transfer provides a small ΔE_ST_ but rISC follows after a thermally activated rIC step.^39^ Supporting this mechanism, calculations by Northey and Penfold^5^ show that in a related DABNA material nonadiabatic coupling of S_1_ to higher lying singlet states mediates enhanced SOC via vibronic coupling, mainly between S_1_ and T_2_. As there is only one large amplitude vibration (120 meV, 960 cm^–1^) coupling to the electronic excited state in ν-DABNA (wagging motion of the peripheral *N*-biphenyl groups), both models would yield highly temperature-dependent rISC as we show here. We suggest that it is this mode that mediates the spin conversion here. Therefore, we conclude that triplet upconversion in ν-DABNA (and other similar multiresonant systems) is through a rIC upper-triplet state crossing mechanism that gives rise to Δ*E*_ST_ (optically determined) insensitive to the environment, with an invariant rISC rate that remains strongly thermally activated.

Alongside the photophysical analysis, we confirm the detrimental effects of the excimer state at low concentration in HF-type OLEDs. The relatively large increase in EQE at 0.5 wt % ν-DABNA loading and subsequent decrement when this is increased further to 1 wt % strongly indicates the performance-limiting effects of excimer quenching. This realization comes despite a Förster radius analysis showing less complete energy transfer in the higher-performance 0.5 wt % device.

While the FRET efficiency is almost total at 1 wt % doping, this work indicates that structural modification of ν-DABNA to suppress excimer formation at 1 wt % may unlock further performance gains. This may be achieved in future by rigidifying the peripheral phenyl groups that can prevent close approach of nearby molecules.^23,40^ However, as we have found that the vibrational modes of the peripheral groups play a critical role in determining both the FWHM of the emission and the rISC rate, care must be taken when chemically modifying these groups. Introducing more or higher energy vibrational modes will broaden the FWHM, which is highly undesirable. Similarly, modifying the *N*-diphenyl groups with more rigid structures could severely reduce rIC rates making rISC highly inefficient.

## Conclusions

The photophysics of ν-DABNA is surprisingly complex for a rigid molecule with high PLQY and few coupling vibronic modes. Here we show that two main vibrations couple to the electronic states; the low amplitude modes of the peripheral phenyl units dictate the FWHM of the emission band. We see that at RT these low energy modes have high thermal population, which broadens the emission band and gives rise to hot exciton emission. More importantly, a large amplitude mode of 120 meV is associated with the *N*-biphenyl end groups. The large amplitude mode mode likely drives vibronic coupling in the molecule to mediate reverse internal conversion from the lowest triplet states and thus enable rISC. Measurements in both solution and solid state show very strong temperature dependence to the observed DF. At RT, the rISC rate seems virtually independent of extrinsic factors such as polarity and host packing, at ca. 3–6 × 10^5^ s^–1^. At 80 K, all rISC is effectively quenched. Phosphorescence measurements reveal that even at 80 K the phosphorescence emission is still red shifting after 40 ms, revealing a temperature-sensitive ΔE_ST_ that approaches the previously reported Arrhenius-derived value of 70 meV only at 20 K. From all of these observations, we deduce that the rISC mechanism in ν-DABNA is mediated by strongly thermally activated rIC of T_1_ states to T_2_ (or higher) followed by rapid rISC from T_2_ (or higher) to S_1_, in line with the theoretical prediction of Northey and Penfold.^5^ Βoth ISC transitions between S_1_ and these upper triplet states are likely further enhanced at RT through large populations of the first and second excited vibronic sublevels of S_1_, which enable more efficient crossing to these higher level triplet states.

More importantly for OLED applications, we uncover a strong prevalence for ν-DABNA to form excimers. Although weakly coupled with large disorder, these form even at 0.1 wt % ν-DABNA loading in a host—well below the concentrations used in devices. HF-type OLEDs consequently show increased performance of 13.3 and 22.2% for 1 and 0.5 wt % doping, respectively, compared to the single TADF emitter device; the performance diminishes as the ν-DABNA concentration is increased. These excimer states have very low emission and are unveiled as a major route for exciton quenching in all ν-DABNA devices, also contributing to lower-than-expected performance in the hyperfluorescent devices explored here. Förster analysis shows that the concentration needs to be close to 1 wt % ν-DABNA to achieve full energy transfer from the TADF cohost, which will require future molecular design to prevent excimer formation at such concentrations. While small modifications to the ν-DABNA peripheral groups could prevent excimer formation and increase PLQY at increased doping levels, these must be carefully designed to avoid introducing new vibrational modes, which increase the FWHM of the exciton emission band and may interfere with vibronic coupling mediating rIC and so effectively killing rISC.

## Supporting Information Available

Details of experimental procedures, X-ray crystallographic data, additional photophysical and OLED data, and kinetic modeling of photophysical data (PDF).
