# Supplementary material for: Hot Vibrational States in a High-Performance Multiple Resonance Emitter and the Effect of Excimer Quenching on Organic Light-Emitting Diodes
Source: ACS Appl Mater Interfaces. 2021 Feb 8;13(7):8643–55. doi: 10.1021/acsami.0c20619 (PMC8023512; doi:10.1021/acsami.0c20619)
Supplement: Supplementary file 1 — am0c20619_si_001.pdf [file am0c20619_si_001.pdf]

# Supporting Information

# Hot vibrational states in high performance multiple resonance emitter and the effect of excimer quenching on organic light-emitting diodes.

Kleitos Stavrou,[†] Andrew Danos,[†] Toshiki Hama,[ζ] Takuji Hatakeyama[ζ], and Andrew Monkman[*,†]

ζ Department of Chemistry, School of Science and Technology, Kwansei Gakuin University, Sanda, Japan.

† Department of Physics, Durham University, South Road, Durham, DH1 3LE, United Kingdom.

***Corresponding Author:***

***Prof Andrew Monkman***

a.p.monkman@durham.ac.uk

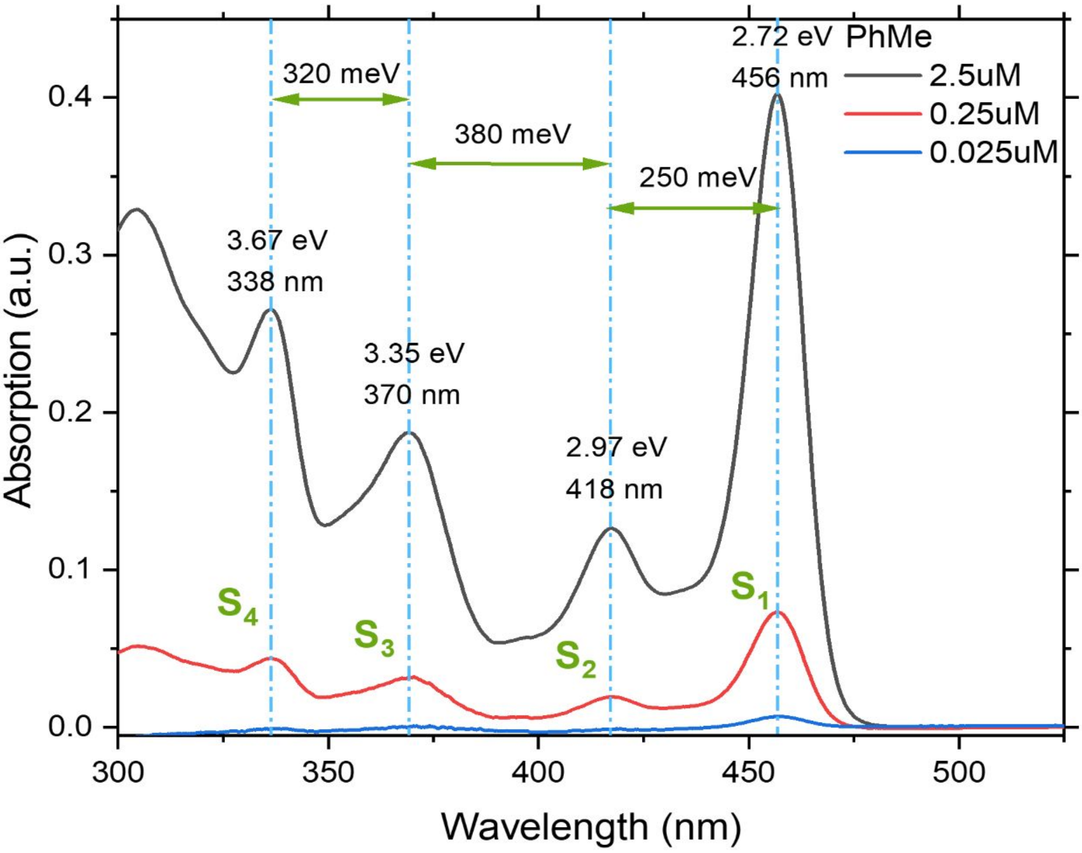


**Figure S1.** Assignment of bands in the absorption spectrum of ν-DABNA in toluene solution, 2.5 μM to 0.025 μM concentrations.

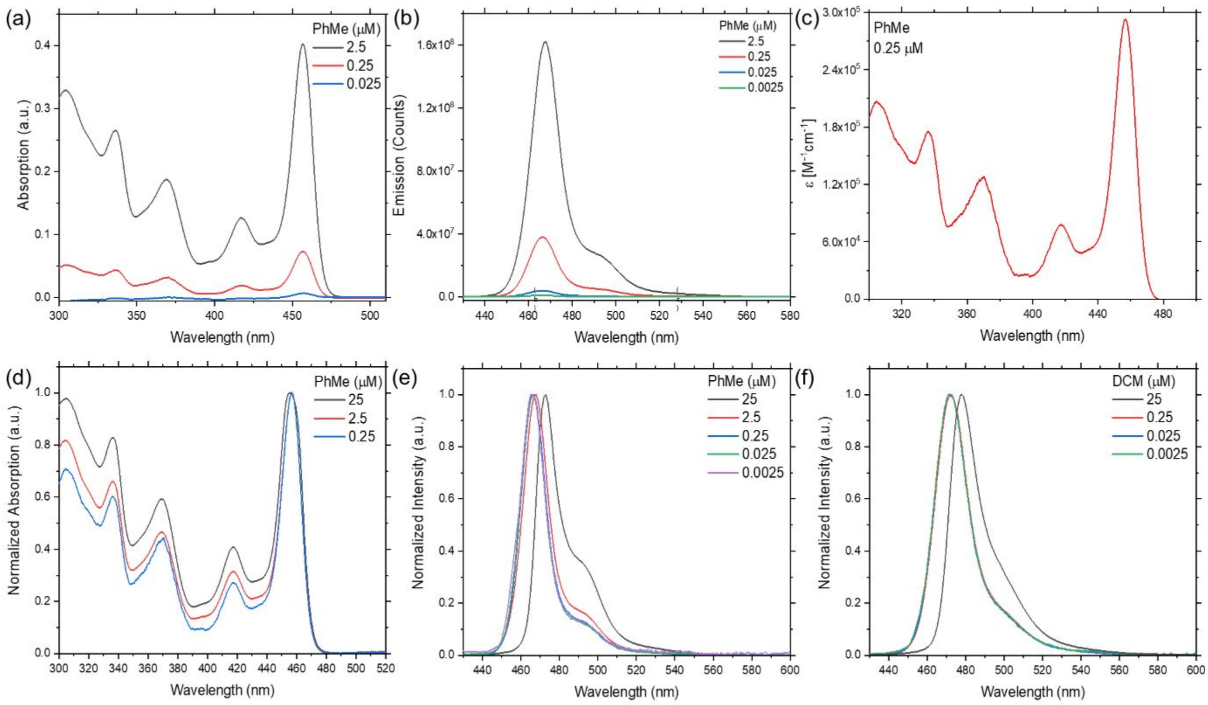


**Figure S2.** Absolute (a), (b) and normalised (d), (e) absorption and emission spectra, respectively, of ν-DABNA in toluene solvent and (f) DCM solvent, at different concentrations. (c) Extinction coefficient calculated at 250 nM, in toluene solvent, is also given. Excitation at 370 nm.

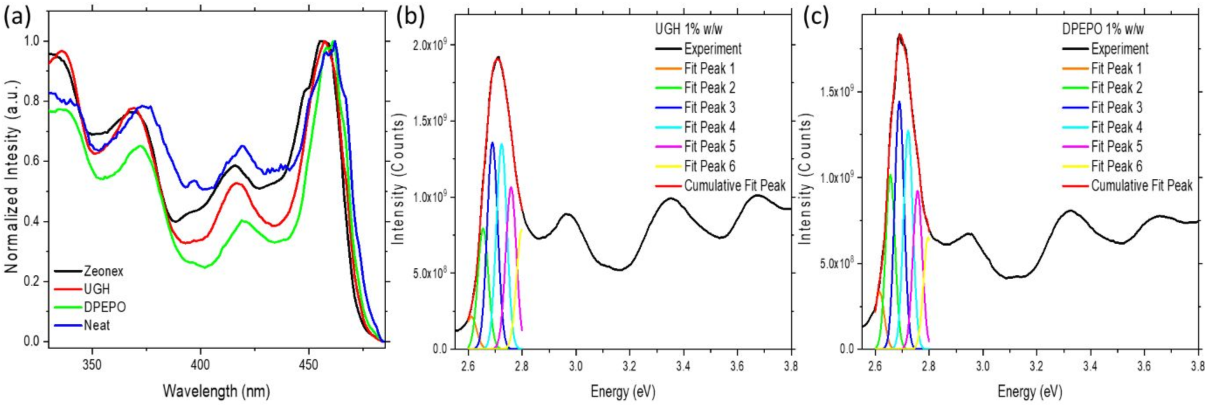


**Figure S3.** (a) Excitation profiles of ν-DABNA in various host matrices and neat film (collection at 500 nm). Multiple Gaussian peak fitting of the leading component (FWHM tied) in (b) UGH and (c) DPEPO matrix.

| Gaussian Peak (Mode) No | | 1 | 2 | 3 | 4 | 5 | 6 |
|---|---|---|---|---|---|---|---|
| **UGH** | $X_C$ (eV) | 2.615 | 2.654 | 2.688 | 2.723 | 2.758 | 2.798 |
| | A ($X10^7$) | 1.05 | 3.89 | 6.66 | 6.61 | 5.22 | 3.86 |
| | FWHM (eV) | 0.046 | 0.046 | 0.046 | 0.046 | 0.046 | 0.046 |
| Energy Difference Between Modes (meV) | | | 39 | 34 | 35 | 35 | 40 |
| **DPEPO** | $X_C$ (eV) | 2.615 | 2.655 | 2.689 | 2.721 | 2.757 | 2.795 |
| | A ($X10^7$) | 1.44 | 4.41 | 6.26 | 5.53 | 4.01 | 2.83 |
| | FWHM (eV) | 0.046 | 0.046 | 0.046 | 0.046 | 0.046 | 0.046 |
| Energy Difference Between Modes (meV) | | | 40 | 34 | 32 | 36 | 38 |

**Table S1.** Excitation profile, leading component, Gaussian fitting parameters of vDABNA in UGH and DPEPO matrix.

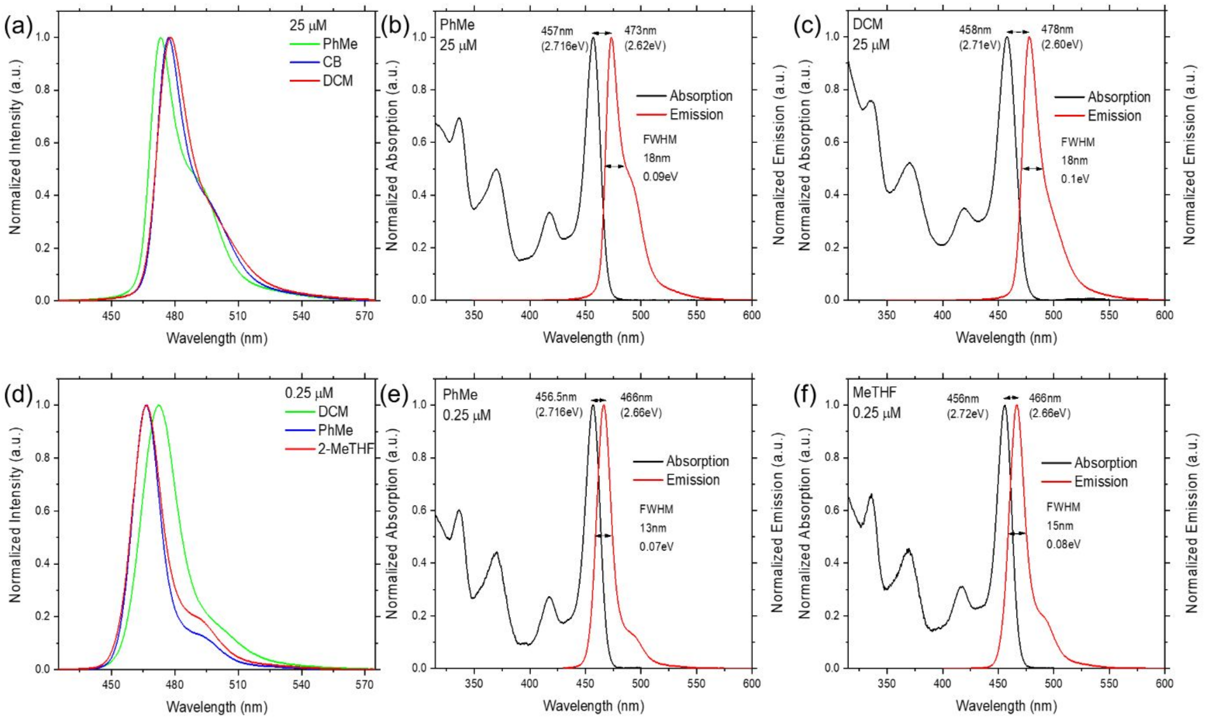


**Figure S4.** Solvatochromism of v-DABNA at (a), (b), (c) 25μM and (d) 0.25μM concentration, in various solvents. The molecule has different degree of solubility depending on the solvent polarity. (e), (f) Effect of the different solvent on the half width of the main emission band is also shown. Excitation at 370 nm.

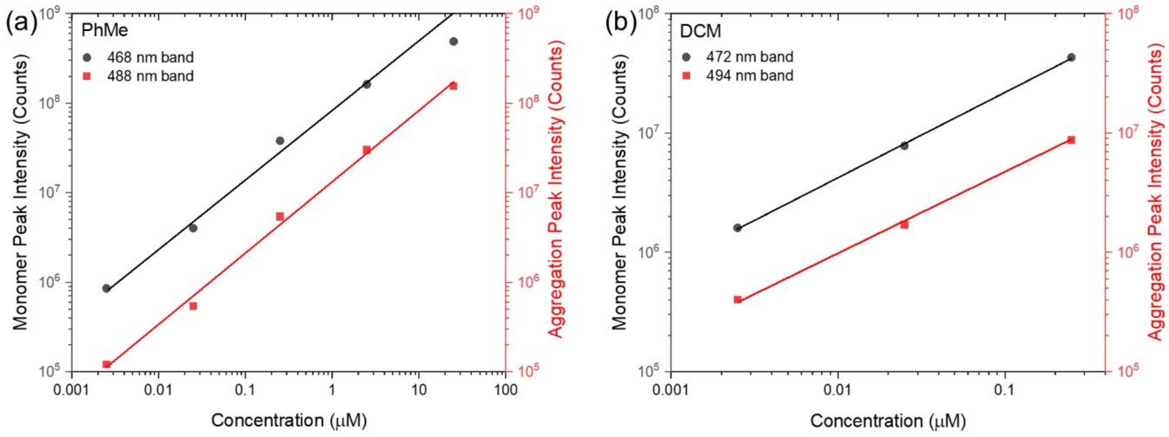


**Figure S5.** Linear fitting of $S_1$-$S_0$ transition peak (black dots) and aggregation related emission peak (red squares) intensity relative to concentration of v-DABNA in (a) toluene and (b) DCM solution.

| v-DABNA PhMe Concentration (μM) | 0.0025 | 0.025 | 0.25 | 2.5 | 25 |
|---|---|---|---|---|---|

| Peak number | 1st | 2nd | 1st | 2nd | 1st | 2nd | 1st | 2nd | 1st | 2nd |
|---|---|---|---|---|---|---|---|---|---|---|
| Peak Wavelength (nm) | 466 | 487 | 466 | 487 | 466 | 488 | 468 | 488 | 473 | 488 |
| Peak intensity (counts) | 8.5E5 | 1.2E5 | 4E6 | 5.4E5 | 3.8E7 | 5.4E6 | 1.6E8 | 3E7 | 3.9E8 | 1.5E8 |

**Table S2.** Comparison of the two emission bands, $S_1$-$S_0$ transition (1st) and aggregation related (2nd), in different concentrations in PhMe.

| v-DABNA DCM Concentration (μM) | 0.0025 | | 0.025 | | 0.25 | | 25 | |
|---|---|---|---|---|---|---|---|---|
| Peak number | 1st | 2nd | 1st | 2nd | 1st | 2nd | 1st | 2nd |
| Peak Wavelength (nm) | 472 | 494 | 472 | 494 | 472 | 494 | 478 | 497 |
| Peak intensity (counts) | 1.6E6 | 4E5 | 7.8E6 | 1.7E6 | 4.3E7 | 8.7E6 | - | - |

**Table S3.** Comparison of the two emission bands, $S_1$-$S_0$ transition (1st) and aggregation related (2nd), in different concentrations in DCM.

| Solvent at 20°C | Viscosity (cp) | Dielectric Constant |
|---|---|---|
| **MeCN** | 0.37 | 37.5 |
| **DCM** | 0.43 | 9.08 |
| **2-MeTHF** | 0.46 | 6.97 |
| **CB** | 0.8 | 5.62 |
| **PhMe** | 0.59 | 2.38 |

**Table S4.** Physical properties of solvents.

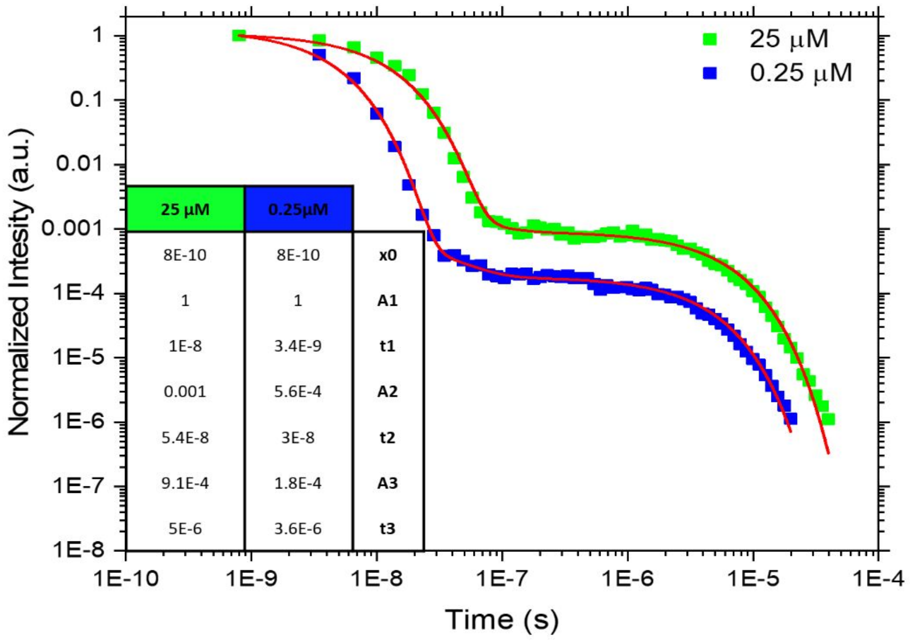


**Figure S6.** Multi-exponential fitting of Time-Resolved PL decays of v-DABNA in toluene solution at 0.25μM and 25μM concentration, at RT. Inset: lifetime fitting results, in nanoseconds. Excitation at 355 nm.

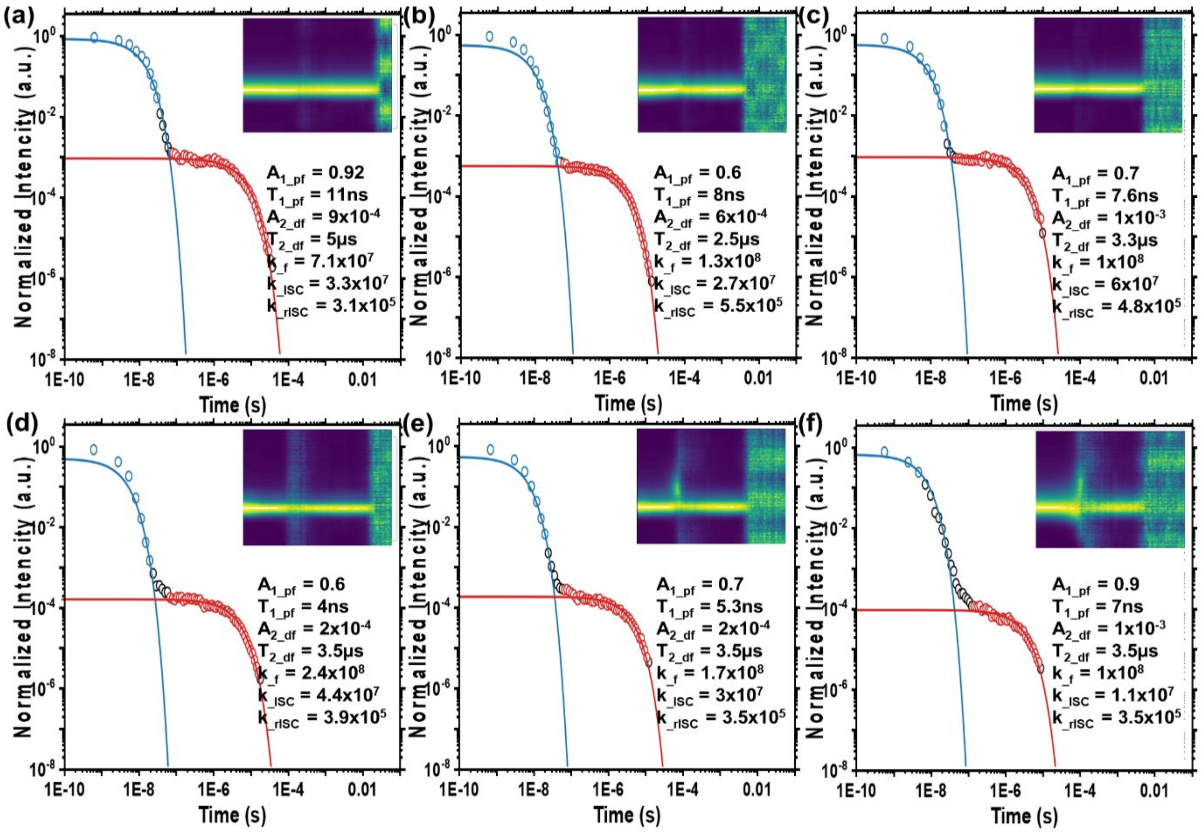


**Figure S7.** Time-Resolved PL decays and kinetic modelling results of v-DABNA in (a) PhMe solution 25μM, (b) CB solution 25μM, (c) DCM solution 25μM, (d) PhMe solution 0.25μM, (e) 2-MeTHF solution 0.25μM and (f) MeCN solution 0.25μM concentration, at 298K. Inset graphs: contour plots of Time-Resolved PL. Excitation at 355 nm.

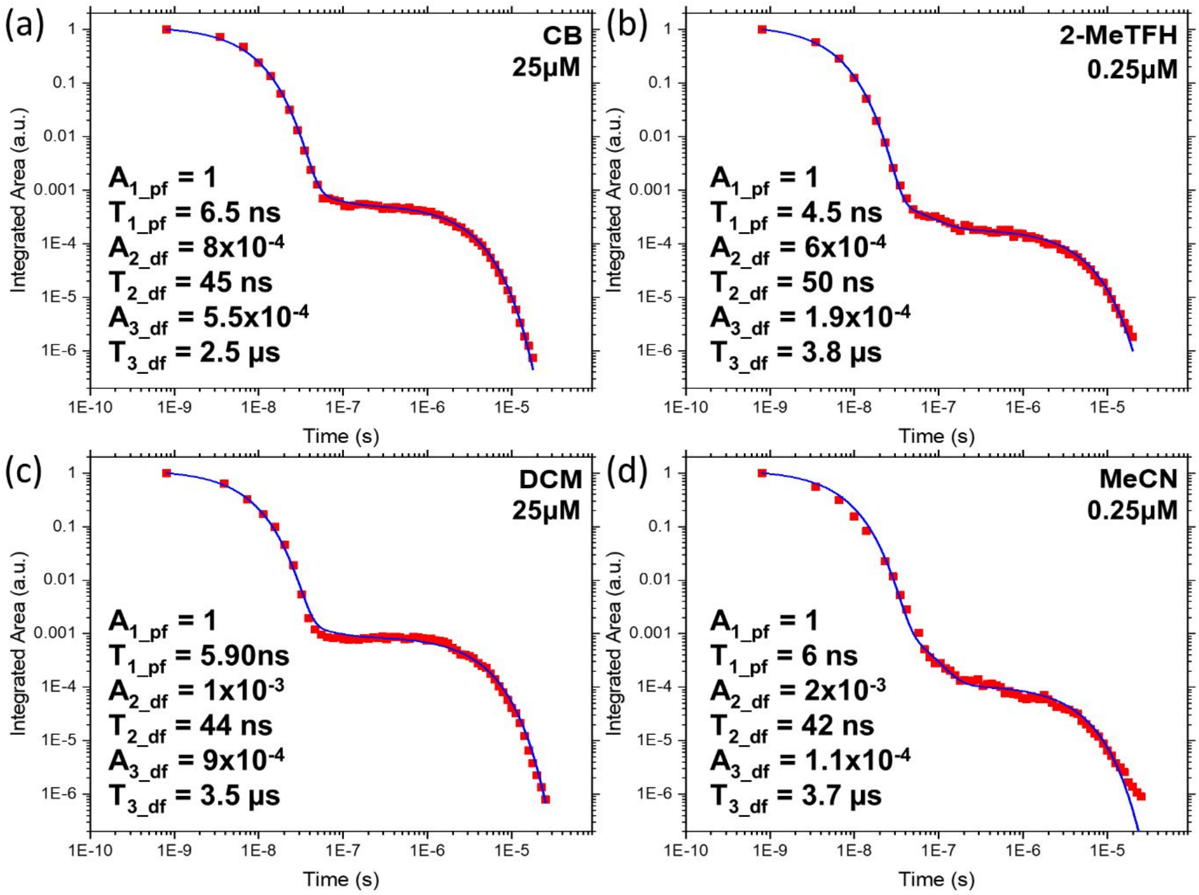


**Figure S8.** Multi-exponential fitting of Time-Resolved PL decays of v-DABNA in (a) CB solution 25µM, (b) 2-METHF solution 0.25µM, (c) DCM solution 25µM and (d) MeCN solution 0.25µM concentration, at RT. Inset: lifetime fitting results. Excitation at 355 nm.

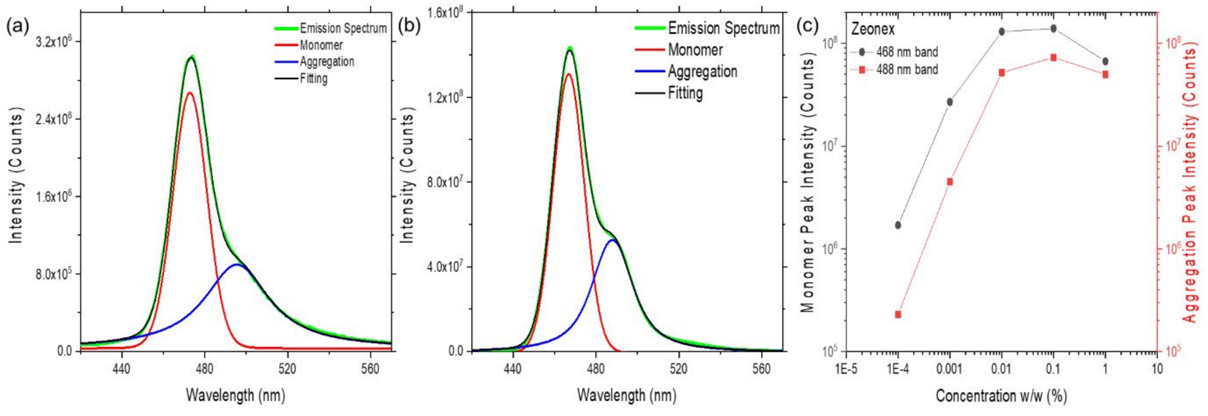


**Figure S9.** Example of deconvoluted emission spectrum of v-DABNA in (a) DEPEPO and (b) Zeonex 1 wt%, using Voigt profile for $S_1$-$S_0$ monomer (red) and aggregation related (blue) transition. (c) Peak intensity of $S_1$-$S_0$ monomer (black) and aggregation related (red) band relative to concentration of v-DABNA in zeonex matrix.

| v-DABNA Zeonex wt (%) | 0.0001 | | 0.001 | | 0.01 | | 0.1 | | 1 | |
|---|---|---|---|---|---|---|---|---|---|---|
| Peak number | 1st | 2nd | 1st | 2nd | 1st | 2nd | 1st | 2nd | 1st | 2nd |
| Peak Wavelength (nm) | 463 | 485 | 464 | 486 | 466.5 | 488 | 468.2 | 488.2 | 470 | 489 |
| Area | 2.9E7 | 6.6E6 | 4.5E8 | 1.5E8 | 2.5E9 | 1.8E9 | 2.5E9 | 2.5E9 | 1.1E9 | 1.9E9 |
| 1st/2nd Area Ratio | 4.4 | | 3 | | 1.3 | | 1 | | 0.6 | |
| Peak intensity (counts) | 1.7E6 | 2.3E5 | 2.7E7 | 4.5E6 | 1.3E8 | 5.2E7 | 1.4E8 | 7.3E7 | 6.7E7 | 5E7 |
| 1st/2nd Peak Ratio | 7.4 | | 6 | | 2.5 | | 1.9 | | 1.3 | |

**Table S5.** Comparison of the two emission bands, $S_1$-$S_0$ monomer ($1^{st}$) and aggregation related ($2^{nd}$), in different concentrations in zeonex matrix.

| v-DABNA UGH wt (%) | 0.01 | | 0.1 | | 1 | | 5 | |
|---|---|---|---|---|---|---|---|---|
| Peak number | 1st | 2nd | 1st | 2nd | 1st | 2nd | 1st | 2nd |
| Peak Wavelength (nm) | 465 | 484 | 467 | 487.5 | 469 | 492.5 | 469 | 497 |
| Area | 1.4E8 | 5E7 | 3.1E8 | 2.2E8 | 6.1E8 | 5.9E8 | 5.5E7 | 1.3E8 |
| 1st/2nd Area Ratio | 2.8 | | 1.4 | | 1.03 | | 0.42 | |
| Peak intensity (counts) | 6.5E6 | 1.3E6 | 1.7E7 | 5E6 | 3.2E7 | 1.2E7 | 3E6 | 2.2E6 |
| 1st/2nd Peak Ratio | 5 | | 3.4 | | 2.6 | | 1.4 | |

**Table S6.** Comparison of the two emission bands, $S_1$-$S_0$ monomer ($1^{st}$) and aggregation related ($2^{nd}$), in different concentrations in UGH matrix.

| v-DABNA DPEPO wt (%) | 0.1 | | 1 | | 5 | |
|---|---|---|---|---|---|---|
| **Peak number** | 1st | 2nd | 1st | 2nd | 1st | 2nd |
| **Peak Wavelength (nm)** | 473 | 495 | 476 | 497 | 478 | 504 |
| **Area** | 5.5E7 | 5.2E7 | 5.5E7 | 1.5E8 | 2.2E7 | 1.7E8 |
| **1st/2nd Area Ratio** | 1.05 | | 0.37 | | 0.13 | |
| **Peak intensity (counts)** | 2.7E6 | 9E5 | 3E6 | 1.7E6 | 1.6E6 | 1.65E6 |
| **1st/2nd Peak Ratio** | 3 | | 1.8 | | 0.98 | |

**Table S7.** Comparison of the two emission bands, $S_1$-$S_0$ monomer (1st) and aggregation related (2nd), in different concentrations in DPEPO matrix.

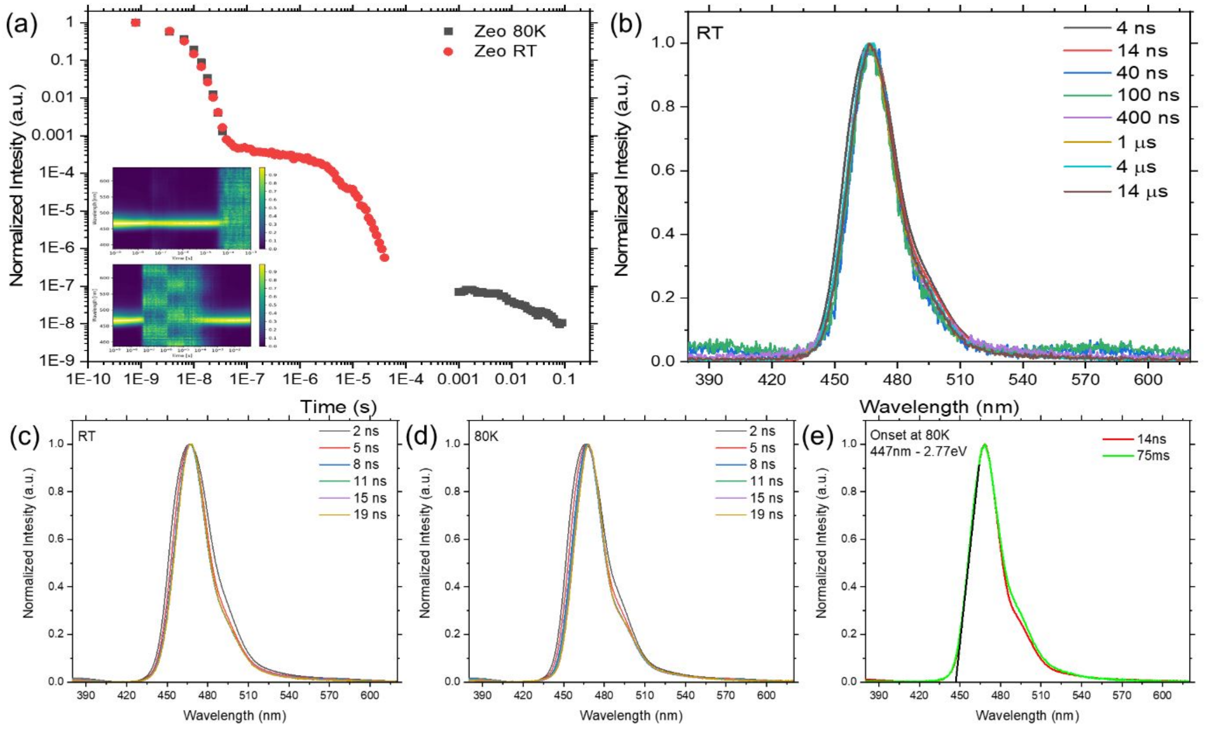


**Figure S10.** (a) Time-Resolved PL decay of ν-DABNA in zeonex matrix, 0.01 wt%, at RT and 80K. Inset graph: contour plots of emission spectrum over time at RT (top) and 80 K (bottom). (b) RT emission spectra at different time delays, analytical early time emission spectra at (c) RT, d) 80 K and (e) prompt and late (phosphorescence) spectra at 80K. Excitation at 355 nm.

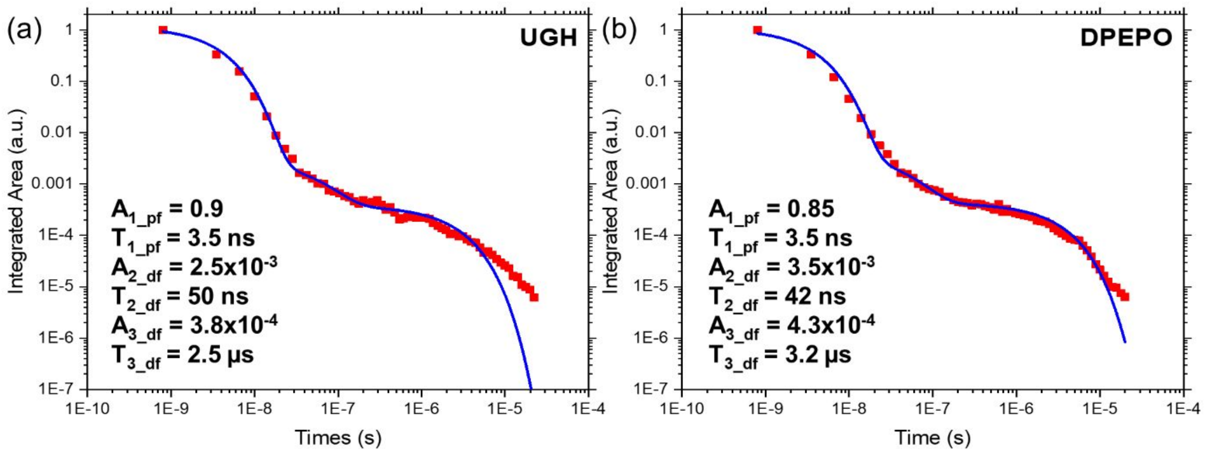


**Figure S11.** Multi-exponential fitting of Time-Resolved PL decays of ν-DABNA, 1 wt%, in (a) UGH and (b) DPEPO matrix, at RT. Inset: fitting results with lifetimes of 3.5 ns and 40-50 ns for the prompt fluorescence and excimer respectively and 2.5 - 3.2 μs for the DF, showing consistency between the two hosts. Excitation at 355 nm.

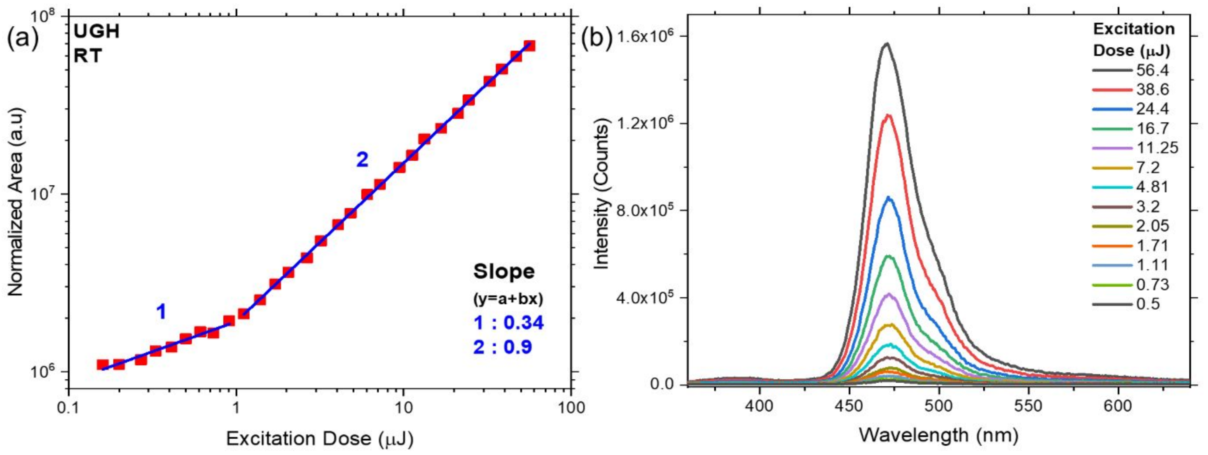


**Figure S12.** (a) Fluence dependence of the delayed emission of 1 wt% ν-DABNA in UGH matrix and (b) spectra, at RT. Excitation at 405 nm.

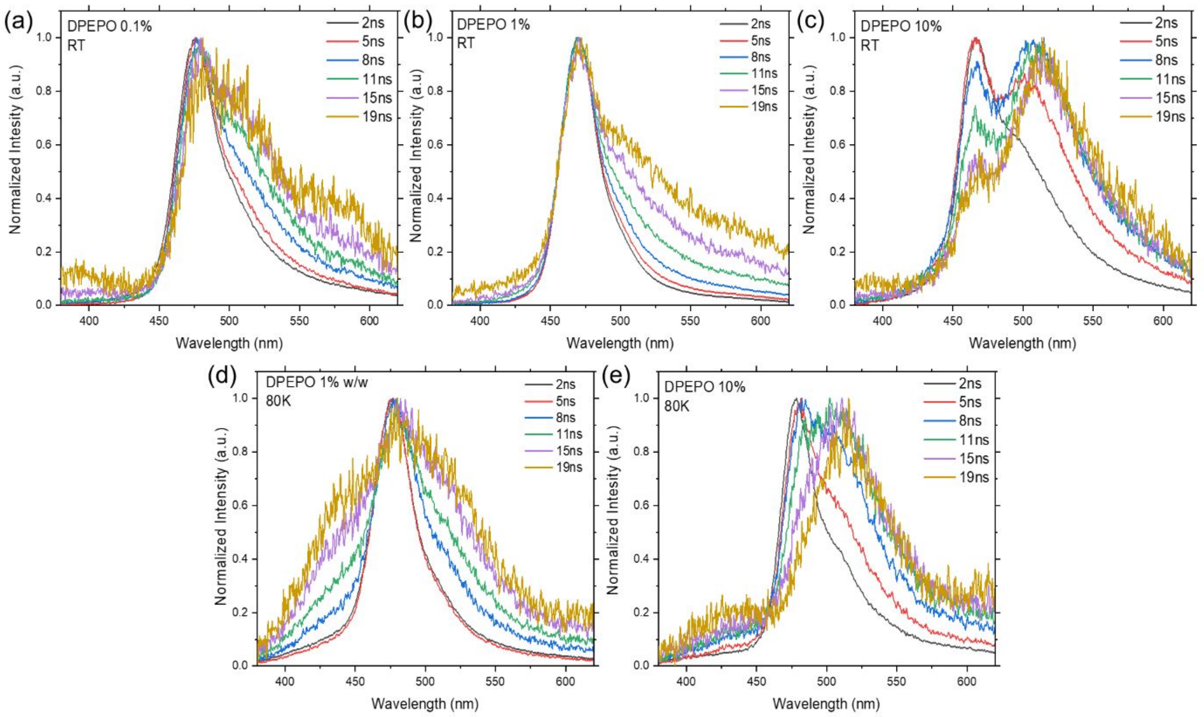


**Figure S13.** Concentration dependence Time-Resolved PL spectra of (a) 0.1 wt% at RT, 1 wt% at (b) RT, (d) 80 K and 10 wt% at (c) RT, (e) 80 K of ν-DABNA in DPEPO matrix. Excitation at 355 nm.

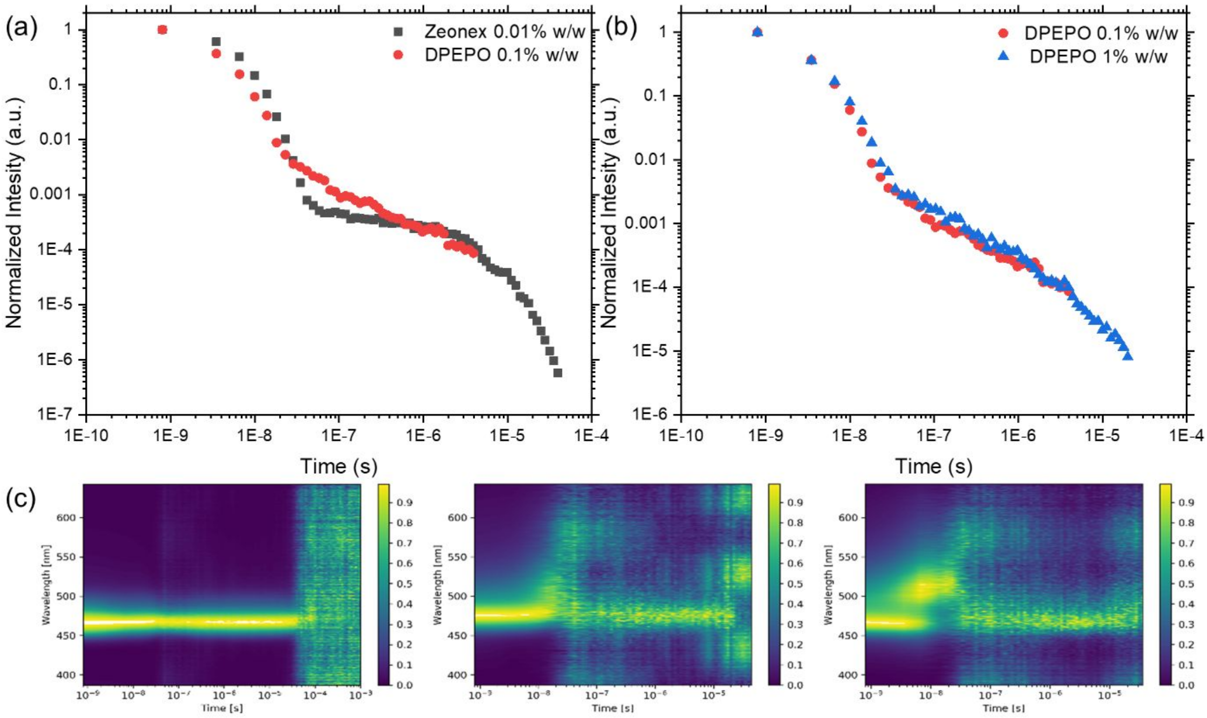


**Figure S14.** (a), (b) Time-Resolved PL decays of ν-DABNA in different host matrices and concentrations. (c) Contour plots of zeonex 0.01 wt%, DPEPO 0.1 wt% and DPEPO 1 wt%, respectively, emission over time showing that the DF ($t > 10^{-7}$ s) arises directly from the exciton state with no excimer contribution. Excitation at 355 nm.

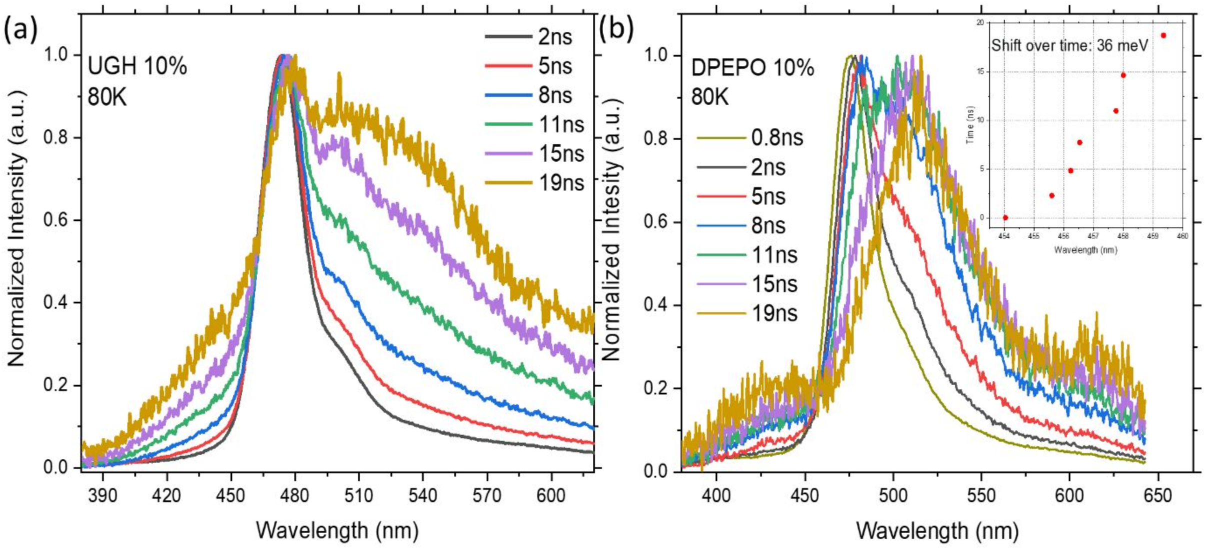


**Figure S15.** 80 K Time-Resolved emission spectra of 10 wt% ν-DABNA in (a) UGH and (b) DPEPO hosts. Inset on DPEPO plot shows linearity and slow rate of spectral relaxation of the excitonic emission band over the first 20 ns. Excitation at 355 nm.

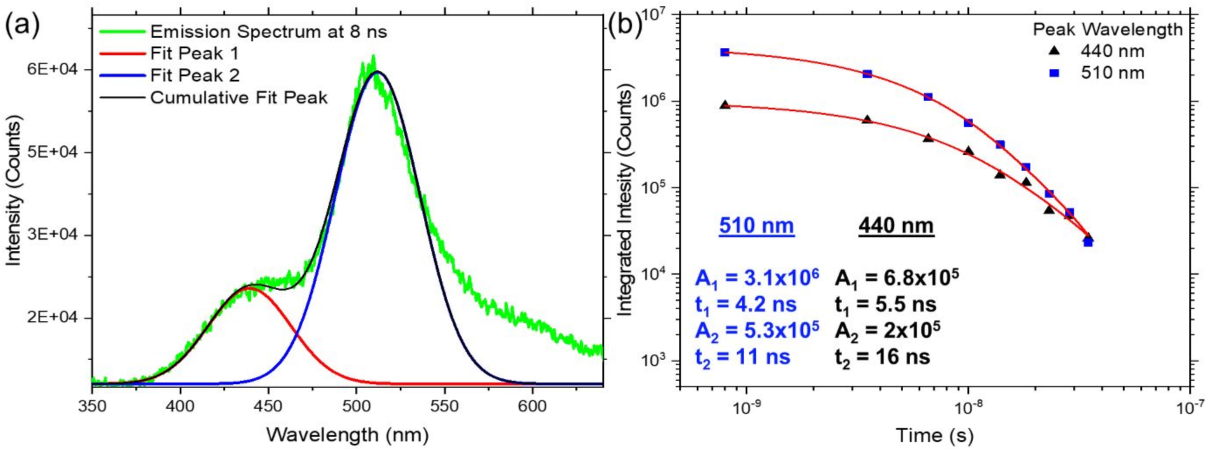


**Figure S16.** (a) Example of a deconvoluted time-resolved emission spectrum of v-DABNA neat film meauresd at 80 K. All time-resolved spectra were deconvoluted and fits made for the respective spectral features (b) to identify the lifetime of each species. A consistent second component with the same lifetime was identified in both decays asigned to an underlying contribution from the exciton decay at 475 nm.

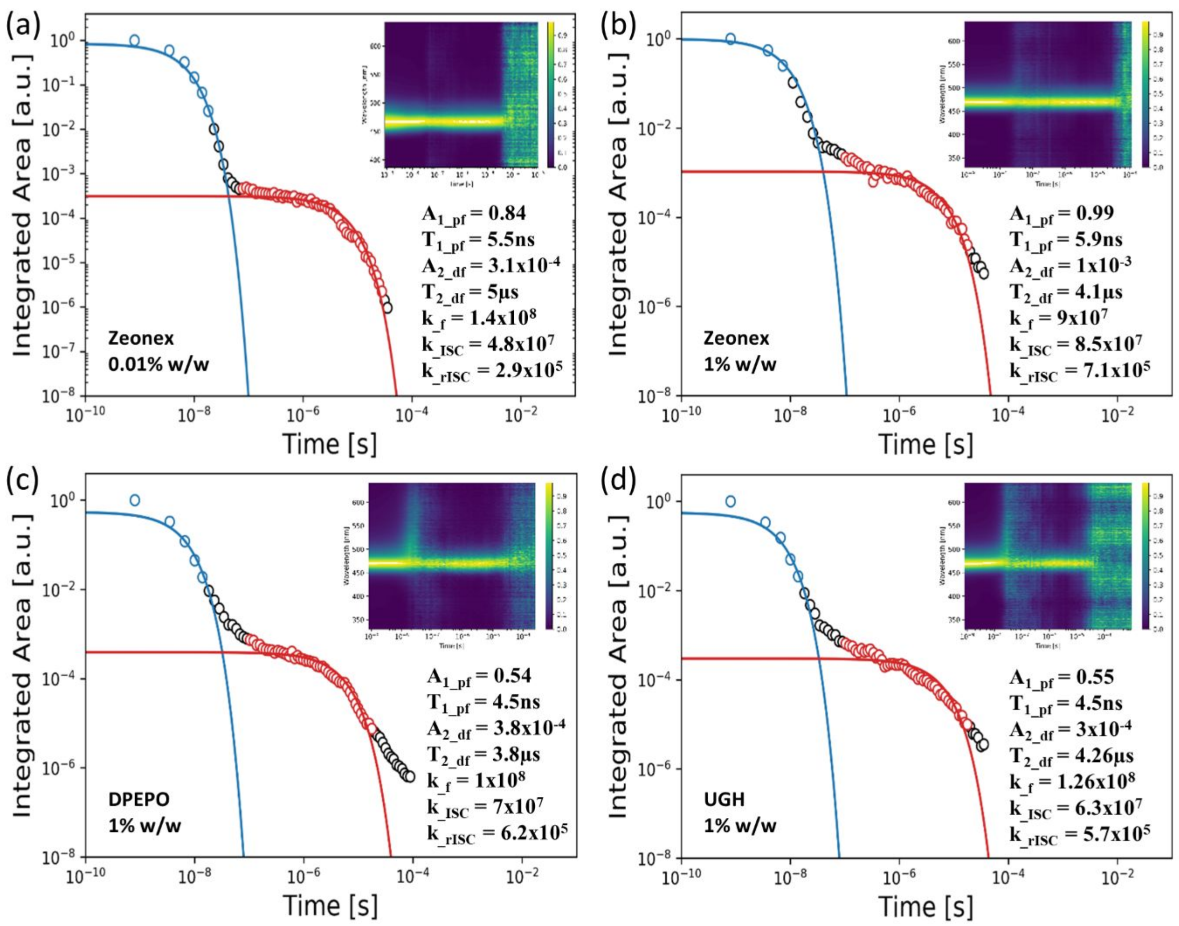


**Figure S17.** Time-Resolved PL decays and kinetic modelling results of v-DABNA in zeonex matrix at (a) 0.01 wt%, (b) 1 wt%, (c) DPEPO matrix at 1 wt% and (d) UGH matrix at 1 wt% concentration, at 298K. Inset graphs: contour plots of Time-Resolved PL. Excitation at 355 nm.

(a)

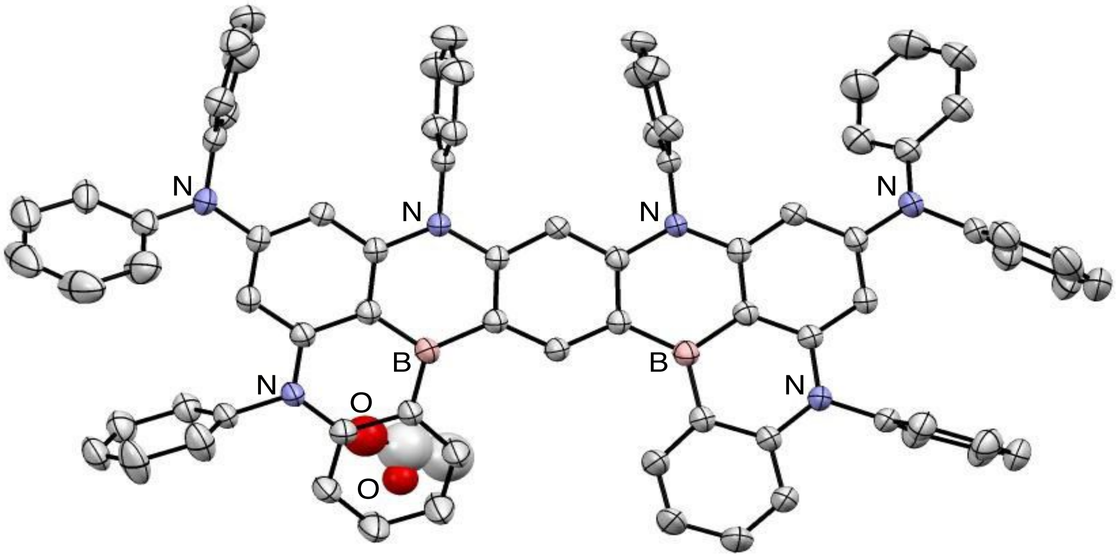


**ν-DABNA**

| | |
|---|---|
| Formula | $C_{78}H_{54}B_2N_6$, 0.5($CH_4O$) |
| Formula Weight | 1112.91 |
| Temperature, K | 150 |
| Wavelength, Å | 0.71075 |
| Crystal System | triclinic |
| Space Group | *P* –1 |
| *a*, Å | 11.064(3) |
| *b*, Å | 15.410(5) |
| *c*, Å | 18.784(5) |
| *β*, deg | 92.680(4) |
| Volume, Å$^3$ | 2983.8(15) |
| *Z* | 2 |
| Density$_{calcd}$, g cm$^{-3}$ | 1.239 |

| | |
|---|---|
| Abs. Coefficient, cm$^{-1}$ | 0.072 |
| F(000) | 1166.0 |
| Crystal Size, mm$^3$ | 0.30, 0.30, 0.30 |
| $2\theta_{min}$, $2\theta_{max}$, deg | 3.013, 24.999 |
| Index Ranges | $-13 \le h \le 13$ |
| | $-18 \le k \le 18$ |
| | $-22 \le l \le 22$ |
| Reflections (unique) | 10449 |
| Reflections ($I$>2.0σ($I$)) | 7919 |
| Parameters | 795 |
| GOF on $F^2$ | 1.093 |
| $R_1$ ($I$>2.0σ($I$)) | 0.0880 |
| $R$, w$R_2$ (all data) | 0.1789 |
| Largest diff peak and hole, e, Å$^{-3}$ | 0.316, –0.230 |

(b)

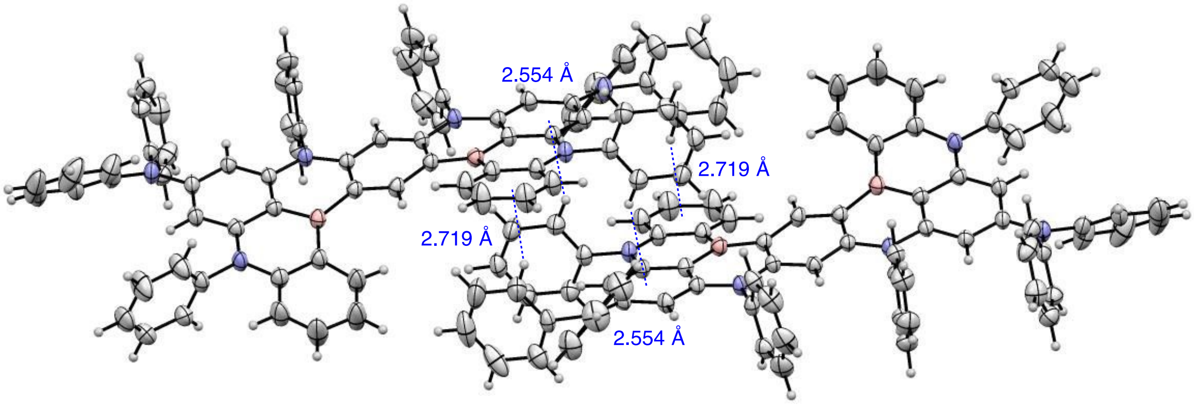

(c)

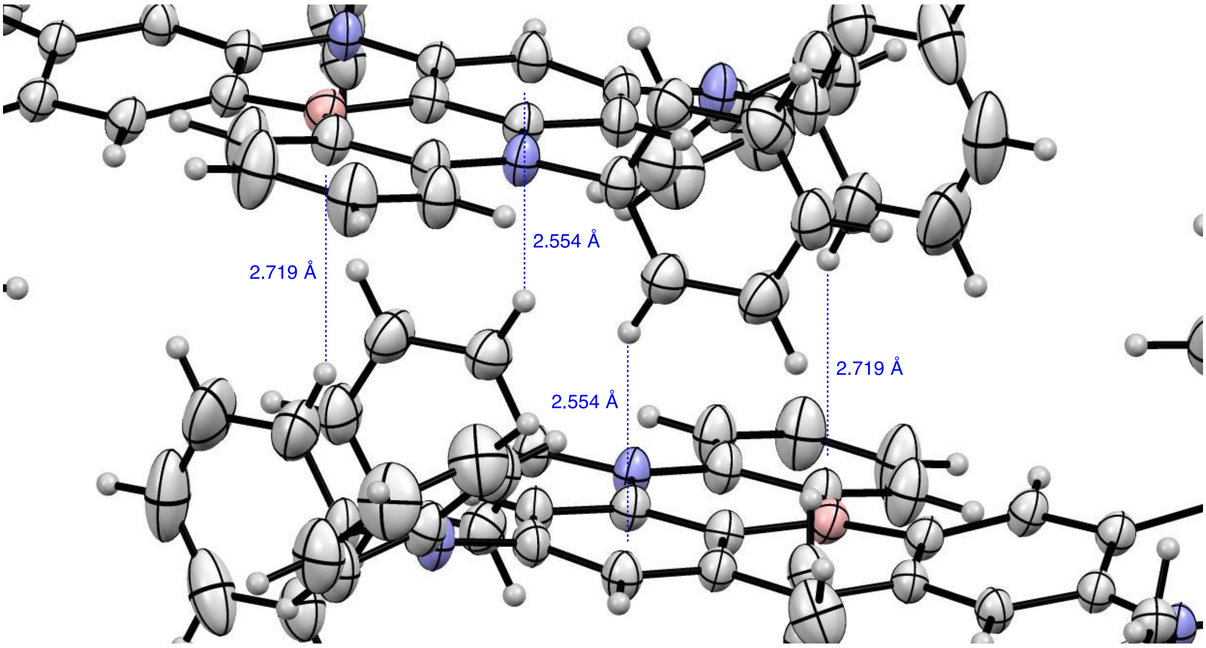


**Figure S18.** (a) X-ray structure of v-DABNA molecule (including one MeOH solvent molecule). (b) Molecular crystal packing indicating short range intermolecular distances (below Van der Waals radii) in cyan. MeOH solvent molecule is omitted for clarity. (c) Expanded view of (b).

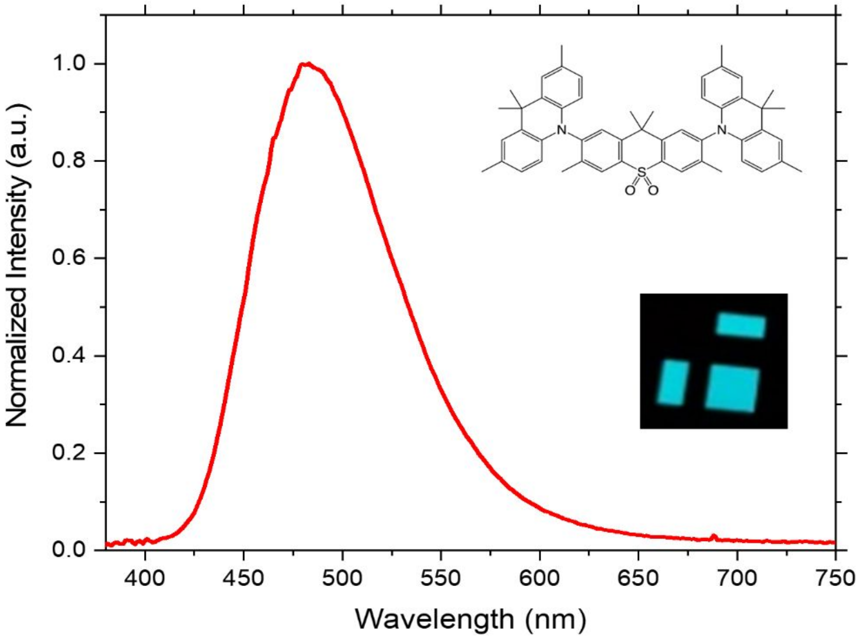


**Figure S19**. OLED's electroluminescence spectra for 35 wt% concertation of TADF emitter in DPEPO host. Inset: TADF emitter structure and operating cyan device.

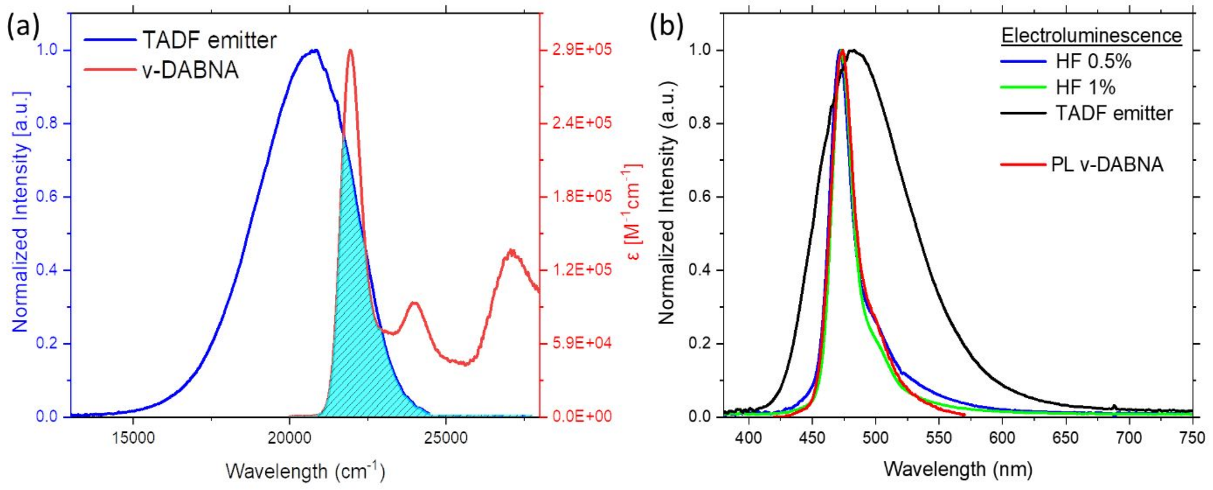


**Figure S20.** Förster radius analysis. (a) Calculated overlap between normalized donor emission spectra and extinction coefficient of the acceptor. (b) Comparison of TADF only and HF OLED devices EL spectra. PL spectra of v-DABNA for comparison.

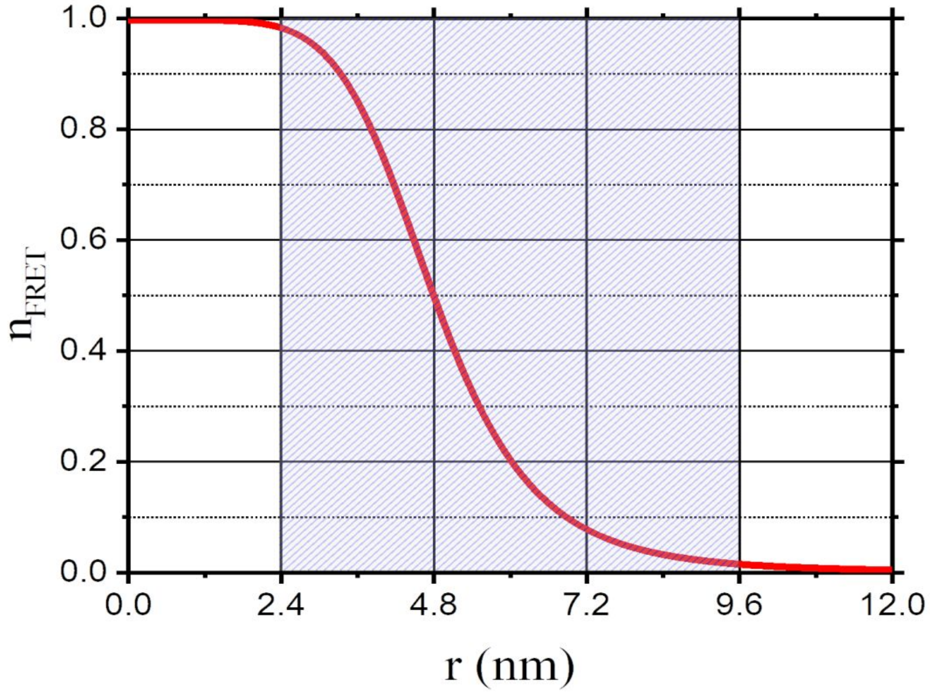


**Figure S21**. FRET efficiency ($n_{FRET}$) as a function of D-A distance (r).[1]

## References

1 Medintz, I.; Hildebrandt, N. FRET - Förster Resonance Energy Transfer. *Wiley Online Lib.* **2013**. https://doi.org/10.1002/9783527656028
